# Virus-associated inflammation imprints an inflammatory profile on monocyte-derived macrophages in the human liver

**DOI:** 10.1172/JCI175241

**Published:** 2025-04-15

**Authors:** Juan Diego Sanchez Vasquez, Shirin Nkongolo, Daniel Traum, Valentin Sotov, Samuel C. Kim, Deeqa Mahamed, Aman Mehrotra, Anjali Patel, Diana Y. Chen, Scott Fung, Anuj Gaggar, Jordan J. Feld, Kyong-Mi Chang, Jeffrey J. Wallin, Ben X. Wang, Harry L.A. Janssen, Adam J. Gehring

**Affiliations:** 1 Toronto Centre for Liver Disease, Toronto General Hospital Research Institute, and; 2 Schwartz Reisman Liver Research Centre, University Health Network, Toronto, Ontario, Canada.; 3 Department of Internal Medicine IV, University Hospital Heidelberg, Heidelberg, Germany.; 4 German Center for Infection Research (DZIF), partner site Heidelberg, Germany.; 5 Department of Medicine, University of Pennsylvania Perelman School of Medicine, Philadelphia, Pennsylvania, USA.; 6 Medical Research, The Corporal Michael J. Crescenz VA Medical Center, Philadelphia, Pennsylvania, USA.; 7 Princess Margaret Cancer Centre, University Health Network, Toronto, Ontario, Canada.; 8 Gilead Sciences, Foster City, California, USA.; 9 Centre for Advanced Single Cell Analysis, The Hospital for Sick Children, Toronto, Canada.; 10 St. Michael’s Hospital, Toronto, Canada.; 11 Department of Gastroenterology and Hepatology, Erasmus MC University Medical Center, Rotterdam, Netherlands.; 12 Department of Immunology, University of Toronto, Toronto, Canada.

**Keywords:** Hepatology, Immunology, Infectious disease, Hepatitis, Macrophages

## Abstract

Chronic liver injury triggers the activation and recruitment of immune cells, causing antigen-independent tissue damage and liver disease progression. Tissue inflammation can reshape macrophage composition through monocyte replacement. Replacement of tissue macrophages with monocytes differentiating in an inflammatory environment can potentially imprint a phenotype that switches the liver from an immune-tolerant organ to one predisposed to tissue damage. We longitudinally sampled the liver of patients with chronic hepatitis B who had active liver inflammation and were starting antiviral therapy. Antiviral therapy suppressed viral replication and liver inflammation, which coincided with decreased myeloid activation markers. Single-cell RNA-Seq mapped peripheral inflammatory markers to a monocyte-derived macrophage population, distinct from Kupffer cells, with an inflammatory transcriptional profile. The inflammatory macrophages (iMacs) differentiated from blood monocytes and were unique from macrophage found in healthy or cirrhotic liver. iMacs retained their core transcriptional signature after inflammation resolved, indicating inflammation-mediated remodeling of the macrophage population in the human liver that may affect progressive liver disease and immunotherapy.

## Abstract 2

A unique population of monocyte-derived macrophages was identified in Chronic hepatitis B patients with an inflammatory profile which may be responsable for disease progression by regulating adaptive immunity.

## Graphical abstract

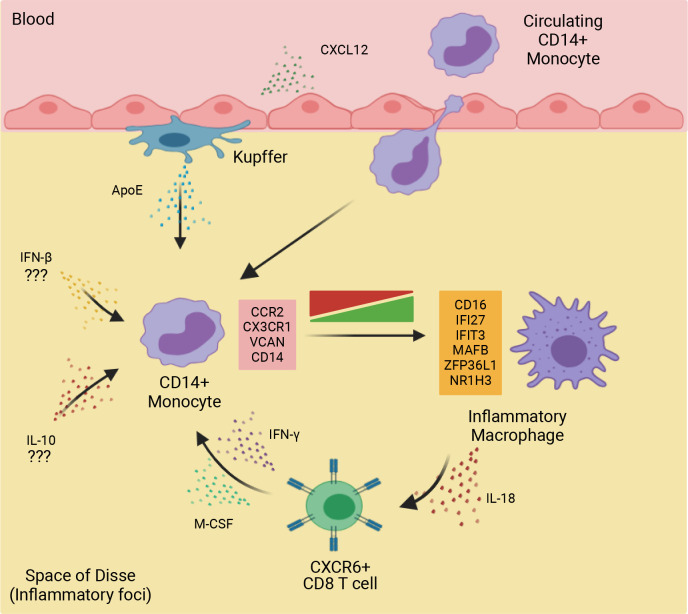

## Introduction

Macrophages play a pivotal role in tissue immunity, representing one of the first lines of defense against pathogens. These cells regulate tissue damage via pathogen clearance and secretion of cytokines, chemokines, and growth factors to avoid unchecked inflammation (1). The macrophage pool is highly heterogeneous, consisting mainly of self-renewing, embryo-derived macrophages that seed the organs during fetal development and monocyte-derived macrophages recruited from the blood upon tissue damage or infection (2, 3). Mouse studies have revealed that monocyte recruitment and imprinting are tissue specific, allowing these cells to differentiate into either long-lived or short-lived macrophages (4–6).

During acute infections, reshaping the macrophage population through an influx of monocyte-derived macrophages improves pathogen clearance and the return to baseline (7). However, more than 800 million people live with chronic liver disease (CLD), which is characterized by persistent inflammation, leading to over 2 million deaths per year from cirrhosis and hepatocellular carcinoma (HCC) (8, 9). In mouse models of liver inflammation,macrophages contribute to disease progression by producing inflammatory cytokines that activate surrounding intrahepatic cells, driving inflammation and tissue injury and causing progressive fibrosis that impairs liver regeneration (8). These inflammatory events can be ameliorated by macrophage depletion in experimental models, underscoring their significant role in tissue regulation (3, 10).

Macrophage-mediated inflammation presents a stark contrast to the steady-state environment of the liver, which is one of tolerance and immune suppression. The tolerogenic environment is maintained via IL-10 production from Kupffer cells (KCs), which are embryonically derived macrophages of the liver (11, 12). This suggests that during CLD, the KC niche is being reshaped by monocyte-derived macrophages that are more inflammatory. This could change the dynamics of immune regulation in the liver and contribute to chronic liver damage, as has been observed in mouse models (10, 13–15).

Understanding how inflammation reshapes liver macrophage composition in humans has been restricted by the ability to collect longitudinal tissue samples within a time frame relevant to inflammation dynamics. However, patients with chronic HBV infection who present to the clinic with active liver damage are started on antiviral therapy. Antiviral therapy suppresses HBV replication and stops liver damage within 6 months. Once started, antiviral treatment can be life-long to maintain suppression of viral replication. Withdrawal of therapy can lead to HBV reactivation and potentially life-threatening liver inflammation that is characterized by a macrophage signature (16–19). Using longitudinal liver fine-needle aspirates (FNAs), we captured dynamic changes in cellular composition and activation during the first 6 months of therapy (20). By combining liver FNA sampling in patients starting antiviral therapy with single-cell RNA-Seq (scRNA-Seq), we achieved a resolution necessary to define heterogeneous macrophage populations and their activation status in the liver of patients with chronic hepatitis B (CHB). This led to the identification of an inflammatory, monocyte-derived macrophage population unique to inflamed livers. These monocyte-derived macrophages were imprinted with a transcriptional profile that was distinguishable even after 4 years of antiviral therapy.

## Results

### Serum markers of myeloid activation map to liver macrophages.

**Figure 1:**
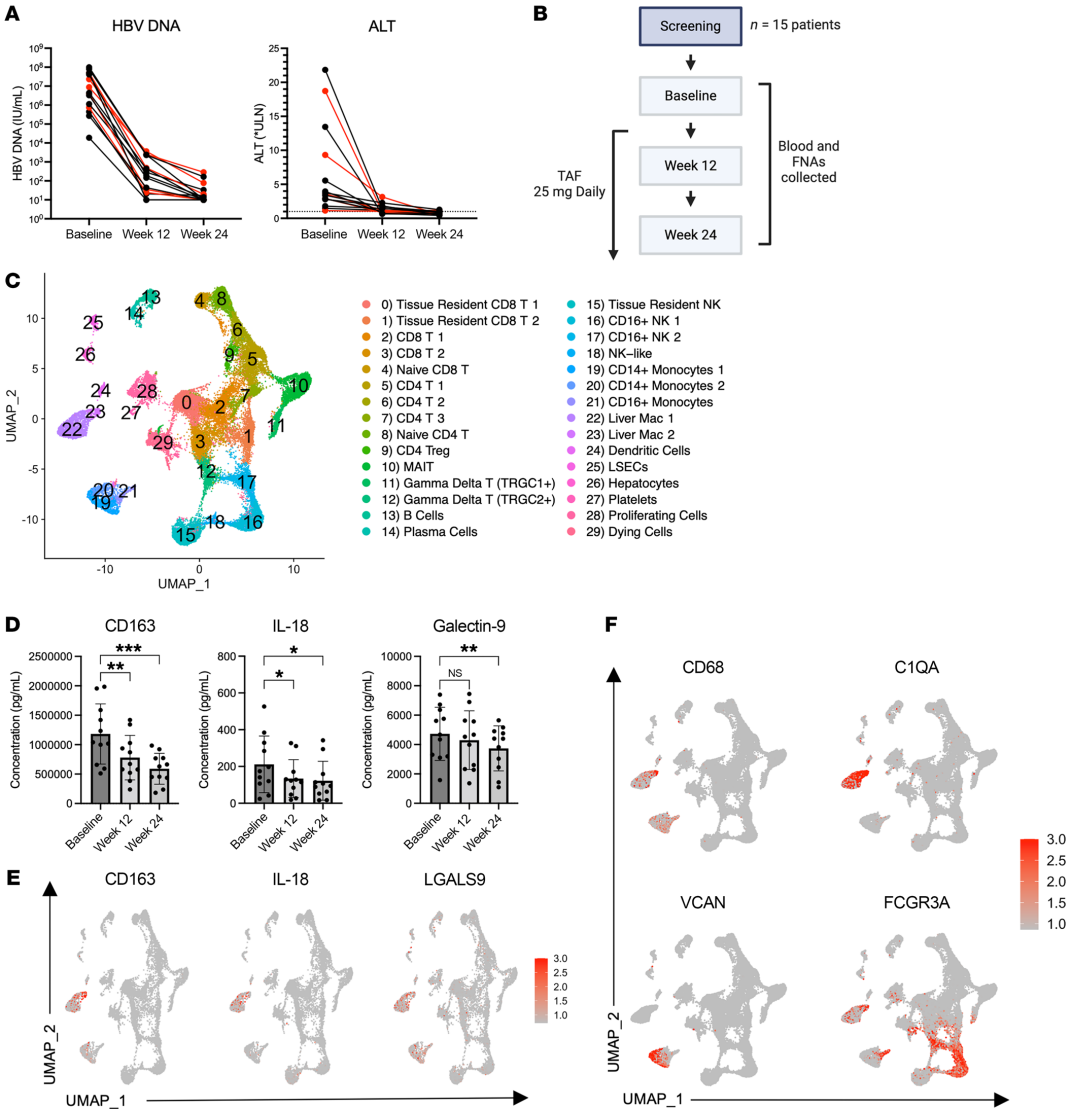
Study design and identification of hepatic cell populations at single-cell resolution from liver FNAs. (**A**) HBV DNA and ALT (displayed as fold change [FC] of the ULN) in blood over time, with patients whose samples were sequenced highlighted in red (*n* = 5). (**B**) Eleven CHB patients with elevated ALT levels started NUC therapy with TAF (25 mg/d). At baseline and after 12 and 24 weeks of therapy, blood and liver FNAs were collected. Longitudinal FNAs from 5 patients were subjected to scRNA-Seq. (**C**) Clustering and annotation of 41,829 cells from human livers for 5 patients across 3 time points (baseline, week 12, and week 24). Uniform manifold approximation and projection (UMAP) dimensionality reduction identified 30 clusters. (**D**) Luminex data from plasma immune profiles of all patients across time. (**E**) Feature plots depicting single-cell gene expression of individual genes detected by the Luminex assay. (**F**) Feature plots depicting single-cell gene expression of liver myeloid cells. *P* values were determined by repeated-measures 1-way ANOVA (*P < 0.05, **P < 0.005, and ***P < 0.001).

To investigate the cellular dynamics of liver inflammation and damage in CLD, we recruited 15 patients with CHB who had ongoing liver damage, as evidenced by elevated levels of serum alanine aminotransferase (ALT) represented as a fold increase over normal values (upper limit of normal [ULN) >1). Patients started antiviral treatment with tenofovir alafenamide (TAF) (25 mg daily), which reduced liver damage and hepatitis B virus (HBV) replication (21) (Figure 1A). We collected liver FNAs at study entry and 12 and 24 weeks after TAF initiation and performed scRNA-Seq in 5 patients (red lines) to link inflammatory biomarkers in the serum to activation of immune cells in the liver (Figure 1B). This allowed us to compare intrahepatic immune profiles during liver injury (baseline) and as inflammation resolved (weeks 12 and 24).

Among the 41,829 cells across the 3 longitudinal time points, we identified 30 distinct cell clusters that had a similar distribution among the different donors (Figure 1C and Supplemental Figure 1, A and B; supplemental material available online with this article; https://doi.org/10.1172/JCI175241DS1). We previously demonstrated that the myeloid activation marker soluble CD163 (sCD163) was significantly increased during liver damage across multiple stages of HBV infection (19). Analysis of serum immune markers in our patient cohort confirmed elevated levels of sCD163 in the serum of patients with hepatitis that significantly declined after 12 weeks of TAF therapy. Consistent with the decline in sCD163, additional myeloid activation markers — IL-18 and galectin-9 — also significantly decreased after starting therapy (Figure 1D). Using the transcriptome data, we mapped these markers to the monocyte and macrophage clusters, defined by the expression of *CD68* (monocyte and macrophage marker), *C1QA/B/C* (macrophage marker), *VCAN* (classical monocyte marker), and *FCGR3A* (nonclassical monocyte marker) (Figure 1, E and F) (22–24). These data validate our previous observations and implicate monocytes and macrophages as potential key players in the pathogenesis of liver injury in CHB (19).

### Inflammatory macrophages are phenotypically distinct from KCs.

**Figure 2:**
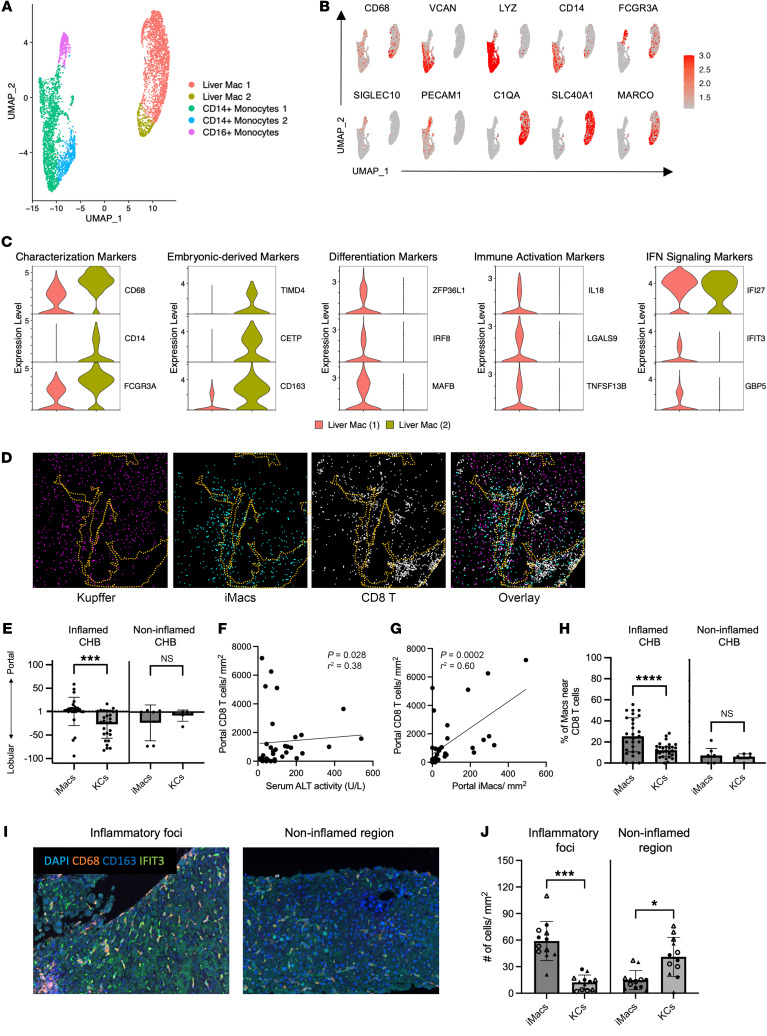
Identification and characterization of myeloid cells during liver inflammation. (**A**) CD68^+^ clusters were reclustered using UMAP dimensionality reduction for 5 patients across 3 time points (baseline, week 12, and week 24). (**B**) Feature plots depicting single-cell gene expression of individual myeloid genes (scale: log-transformed gene expression). (**C**) Violin plots of macrophage-defining genes. All selected genes have an adjusted *P* value of less than 0.05. (**D**) IMC depicting liver macrophages and CD8^+^ T cell colocalization in inflamed livers of patients with CHB. The portal region is outlined by yellow dotted lines. (**E**) Enrichment of liver macrophages depicted by the ratio of the portal divided by the lobular cell count/mm^2^ between inflamed (*n* = 28) and noninflamed (*n* = 6) liver tissue sections. Lobular enrichment values were multiplied by –100. *P* values were determined by a nonparametric Mann-Whitney *U* test (*P < 0.05, **P < 0.005, and ***P < 0.001). (**F** and **G**) Simple correlation analysis between portal CD8^+^ T cells/mm^2^ and (**F**) serum ALT levels and (**G**) iMacs/mm^2^ in patients with CHB. (**H**) Proximity analysis of CD8^+^ T cells and liver macrophages within 15 μm between inflamed (*n* = 28) and noninflamed (*n* = 6) liver tissue sections. *P* values were determined by nonparametric Wilcoxon test (*P < 0.05, **P < 0.005, and ***P < 0.001). (**I**) Multiplex IF images showing liver macrophages in inflamed liver of patients with CHB at inflammatory foci and noninflamed regions (*n* = 4). Original magnification, ×20. (**J**) Quantification of liver macrophages in inflamed liver of patients with CHB at inflammatory foci and noninflammatory regions. *P* values were determined by nonparametric Wilcoxon test (*P < 0.05, **P < 0.005, and ***P < 0.001).

Because serum markers suggested myeloid involvement, CD68^+^ cells were subclustered for further analysis. This led to the identification of 2 classical monocyte clusters (expression of *VCAN*, *LYZ*, and *CD14*), 1 nonclassical monocyte cluster (CD16^+^ monocytes; *FCGR3A* [CD16a]*, SIGLEC10*, and *PECAM1*); and 2 macrophage clusters (Liver Mac 1 and Liver Mac 2; *C1QA* [complement component]; *SLC40A1* [ferroportin]; and *MARCO*) (Figure 2, A and B) (4, 5, 22–25).

Both macrophage clusters expressed *CD68* and *FCGR3A* (CD16a), but Liver Mac 1 could be distinguished by the lack of *CD14* expression (Figure 2C, first column). Liver Mac 2 expressed markers of embryonically derived macrophages such as *TIMD4* (26, 27), *CETP*, and *CD163* (Figure 2C, second panel). In contrast, Liver Mac 1 was enriched for markers of recent monocyte-to-macrophage differentiation (*ZFP36L1*, *IRF8*, and *MAFB*) (4, 5, 28, 29) (Figure 2C, third panel), immune activation (*IL18*, galectin-9 [*LGALS9*], and BAFF [*TNFSF13B]*) (30–32) (Figure 2C, fourth panel), and IFN signaling (*IFI27*, *IFIT3*, and *GBP5*) (33, 34) (Figure 2C, fifth panel). These data demonstrate the coexistence of multiple macrophage populations during active liver damage in patients with CHB. Based on the transcriptional profiles, Liver Mac 1 and Liver Mac 2 are referred to hereafter as inflammatory macrophages (iMacs) and KCs, respectively.

Consistent with the transcriptional differences for each macrophage population, their predicted functional profiles differed by gene set enrichment analysis (GSEA) (35). We performed GSEA between baseline and week 24. We found that processes related to inflammation, IFN signaling, and differentiation were enriched in the iMacs at baseline (Supplemental Figure 2A). KCs were also enriched in IFN signaling and antigen presentation but differed by pathways associated with collagen metabolism and proliferation (Supplemental Figure 2B). These data underscore the heterogeneous nature of macrophages and how they may have unique responses during liver inflammation, despite residing in the same organ.

To validate differences in the transcriptional phenotype at the protein level, we performed imaging mass cytometry (IMC) on biopsies from CHB patients with (*n* = 28) and without (*n* = 6) liver inflammation (Figure 2D and Supplemental Figure 2, C and D). KCs, defined as CD68^+^CD16^+^CD14^+^, were evenly distributed throughout the tissue. In contrast, we found that iMacs characterized as CD68^+^CD16^+^CD14^–^ significantly clustered near the portal regions in inflamed tissues compared with noninflamed tissues (Figure 2E). The number of periportal CD8^+^ T cells significantly correlated with ALT levels (Figure 2F) as well as the iMac frequency in the portal area (Figure 2G). Further analysis showed that iMacs were more likely to be in close physical proximity to CD8^+^ T cells during inflammation compared with KCs (Figure 2H). Localization to the portal area suggests that the iMacs had been recently recruited to the liver. Colocalization with CD8^+^ T cells, which are known to both control HBV replication and cause antigen-independent liver damage, may indicate that iMacs play a central role in the progression of CLD (20).

We further characterized the distribution of liver macrophages using more refined markers. We performed multiplex immunofluorescence (IF) on biopsies from CHB patients with liver inflammation (*n* = 4) and analyzed macrophage content in inflammatory foci and noninflamed regions. As predicted by the scRNA-Seq data, iMacs uniquely expressed IFN-induced protein with tetratricopeptide repeats 3 (IFIT3), whereas CD163 was used to identify KCs (Figure 2C). CD68^+^IFIT3^+^ iMacs were significantly enriched within the inflammatory foci. In contrast, CD68^+^CD163^+^ KCs were dispersed throughout the liver lobule (Figure 2, I and J, and Supplemental Figure 2E) (19). These data support the differential distribution of macrophages in the inflamed liver and suggest they regulate different aspects of liver inflammation.

### iMacs are unique to the inflamed liver.

**Figure 3:**
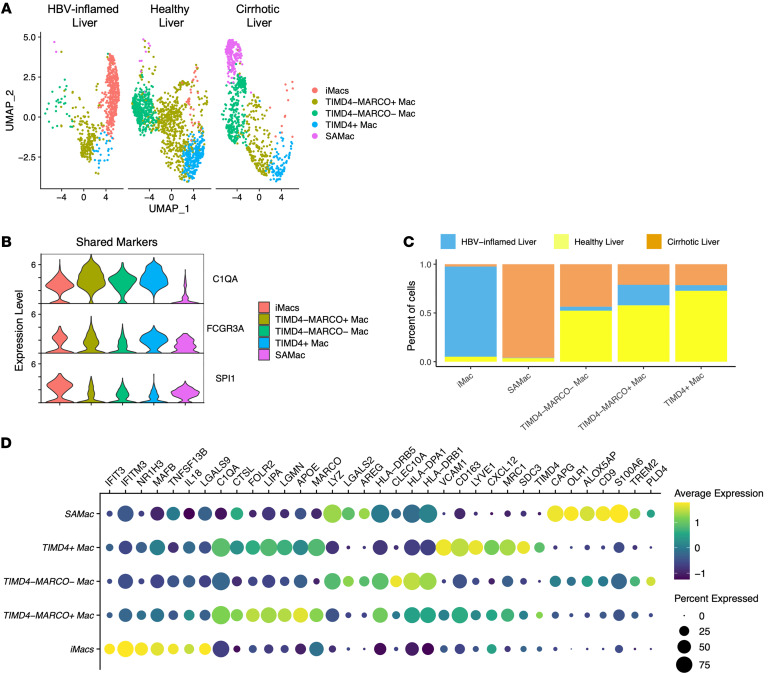
Comparison of iMacs during HBV-related inflammation with healthy and cirrhotic livers at the single-cell level. (**A**) Clustering of macrophages from healthy (*n* = 5), cirrhotic (*n* = 5), and HBV-inflamed (*n* = 5) human livers using UMAP dimensionality reduction by liver condition. Cells from both healthy and cirrhotic livers were obtained from liver tissue digestion and sequenced with 10x Genomics 3′ version 2, which explains why tissue digestion collected more macrophages than with the FNA approach. (**B**) Violin plots of macrophage-shared genes across 3 groups. All selected genes have an adjusted *P* value of less than 0.05. (**C**) Proportions of liver macrophages across 3 groups for each cluster. (**D**) Representation of cluster-defining genes; all selected genes have an adjusted *P* value of less than 0.05.

The IMC and multiplex IF data suggested that iMacs are restricted to patients with active liver inflammation. To confirm this observation at the transcriptional level, we compared macrophages taken from the baseline sample in our study and compared them with macrophages from uninfected healthy and cirrhotic human liver scRNA-Seq datasets. We identified 5 distinct macrophage populations (Figure 3A) (13, 23). All liver macrophages were characterized by the expression of *C1QA*, *SPI1*, and *FCGR3A* (Figure 3B). Since all the other scRNA-Seq datasets were collected from digestion of liver tissue, they recovered a higher number of macrophages that contributed to better-defined clusters compared with liver FNAs. This is likely the reason that our cluster previously defined as KCs revealed 3 distinct sub-macrophage clusters with distinct phenotypes: TIMD4^+^ macrophages, TIMD4^–^MARCO^+^ macrophages, and TIMD4^–^MARCO^–^ macrophages, which were shared among the 3 types of livers (Figure 3C). Scar-associated macrophages (SAMacs) were largely restricted to the cirrhotic liver, whereas iMacs were almost unique to the inflamed liver (Figure 3, A and C) and displayed a distinct transcriptional signature of inflammation and differentiation compared with all other macrophage populations (Figure 3D). These data demonstrate that liver inflammation gives rise to a population of macrophages not found in healthy individuals or cirrhotic livers.

### Trajectory analysis reveals monocyte-to-macrophage differentiation during liver inflammation.

**Figure 4:**
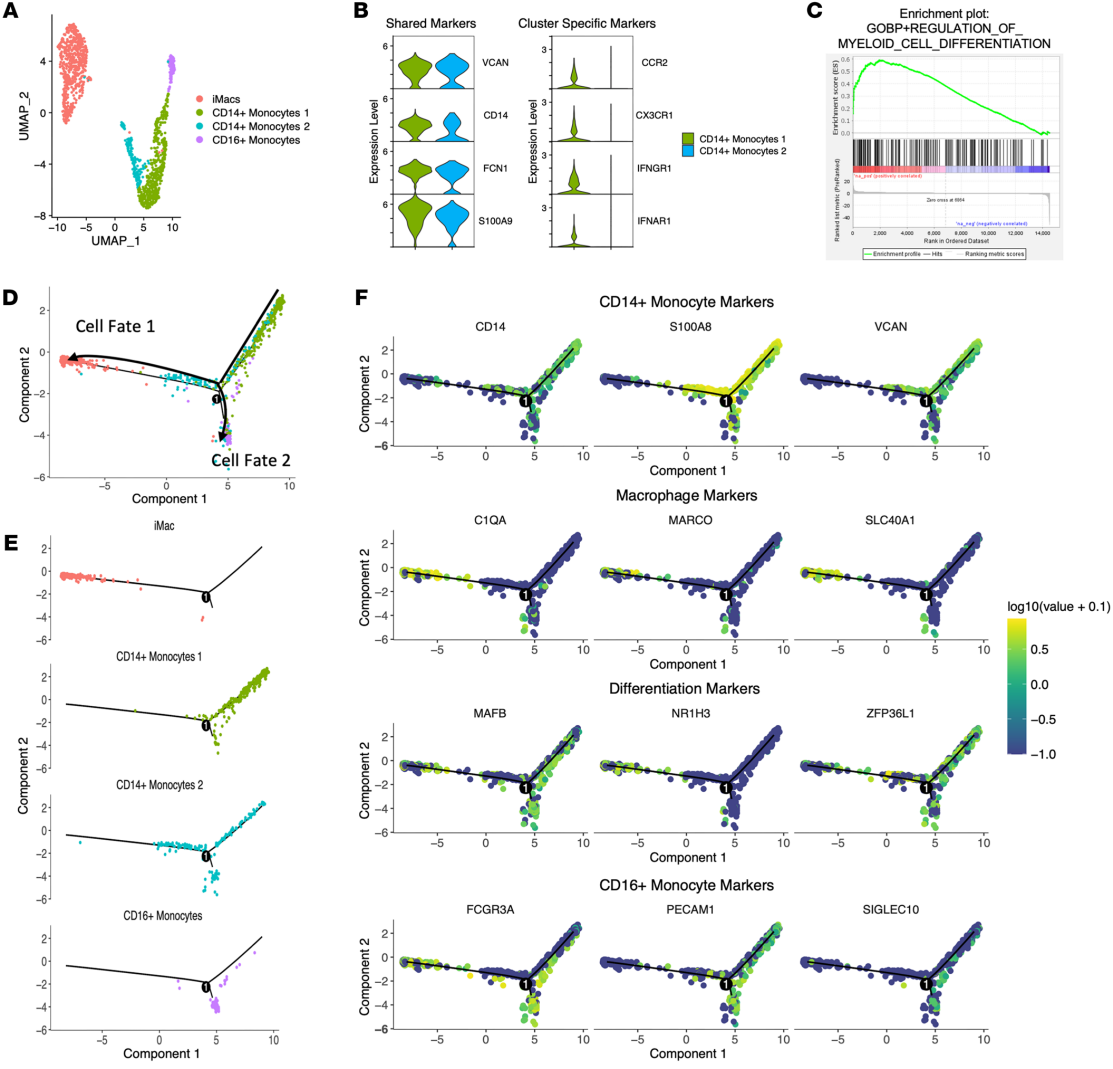
Analysis of monocyte/macrophage differentiation trajectories during liver inflammation in patients with CHB before antiviral treatment. (**A**) iMacs, both CD14^+^ monocytes and CD16^+^ monocytes, were reclustered using Seurat during liver inflammation using UMAP dimensionality reduction. (**B**) Violin plots of CD14^+^ monocyte–defining genes. All selected genes have an adjusted *P* value of less than 0.05. (**C**) Enrichment plot from pathway analysis done on CD14^+^ monocytes at baseline versus week 24 of TAF treatment. Each vertical line represents a differentially expressed gene belonging to the pathway. (**D**) Genes with differential expression between clusters were used to generate hypothetical developmental relationships using the Monocle algorithm. (**E**) Individual clusters along the Monocle trajectory. (**F**) Gene expression of CD14^+^ monocyte–defining (first row), iMac-defining (second row), differentiation-defining (third row), and CD16^+^ monocyte–defining (fourth row) genes along the pseudotime trajectory.

In animal models of liver disease, the KC pool is progressively replaced by infiltrating monocyte-derived macrophages that can be more inflammatory (10). The transcriptional profile of iMacs suggested a similar pathway in the inflamed human liver (Figure 2, C and D). Therefore, we assessed whether CD14^+^ monocytes had gene expression patterns consistent with differentiation into iMacs. Both CD14^+^ monocyte populations (monocytes 1 and monocytes 2) expressed *VCAN*, *CD14*, *FCN1*, and *S100A8*, but were distinguished from one another by the expression of *CCR2*, *CX3CR1*, *IFNGR1*, and *IFNAR1* (Figure 4, A and B). GSEA analysis of cluster CD14^+^ monocytes 1 indicated upregulation of pathways associated with myeloid differentiation during liver inflammation (Figure 4C).

To characterize the developmental relationships between CD14^+^ monocytes, CD16^+^ monocytes, and iMacs, Seurat-defined clusters were subclustered and superimposed on a pseudotime trajectory produced by the Monocle algorithm (36) (Figure 4, A and D). Monocle analysis revealed both CD14^+^ monocyte populations at different points in a successive manner along the same trajectory, representing cells in different states of differentiation, which matched the GSEA prediction (Figure 4D). Furthermore, the trajectory suggested 2 potential cell fates, in which cell fate 1 corresponded to iMacs and cell fate 2 corresponded to CD16^+^ monocytes. The majority of CD14^+^ monocytes 2 oriented toward cell fate 1, implying that the iMacs were derived from CD14^+^ monocytes (Figure 4E).

Gene expression was then plotted as a function of pseudotime to track changes along the trajectory during monocyte-to-macrophage differentiation. *CD14*, *S100A8*, and *VCAN* expression was high in CD14^+^ monocytes 1, lower in CD14^+^ monocytes 2, and absent in the iMacs, suggesting that the cells lost monocyte identity over the course of the trajectory. Loss of monocyte identity was associated with gain of *C1QA*, *MARCO*, and *SLC40A1* in iMacs (Figure 4F). Monocyte markers of differentiation, *MAFB* and *ZFP36L1*, decreased along the path to iMacs, while the macrophage lineage marker *NR1H3* increased (Figure 4F). CD16^+^ monocytes showed progressively increased expression of *FCGR3A*, *PECAM1*, and *SIGLEC10* and retained *MAFB* and *ZFP36L1* expression, suggesting that they could also originate from the differentiation of CD14^+^ monocytes (Figure 4F). This analysis indicates a differentiation pathway from blood monocytes to a transitional CD14^+^ monocyte population before assuming the monocyte-derived macrophage phenotype within the liver.

### Drivers of iMac differentiation are highly expressed in tissue-resident CD8^+^ T cells and KCs in the liver of patients with CHB.

**Figure 5:**
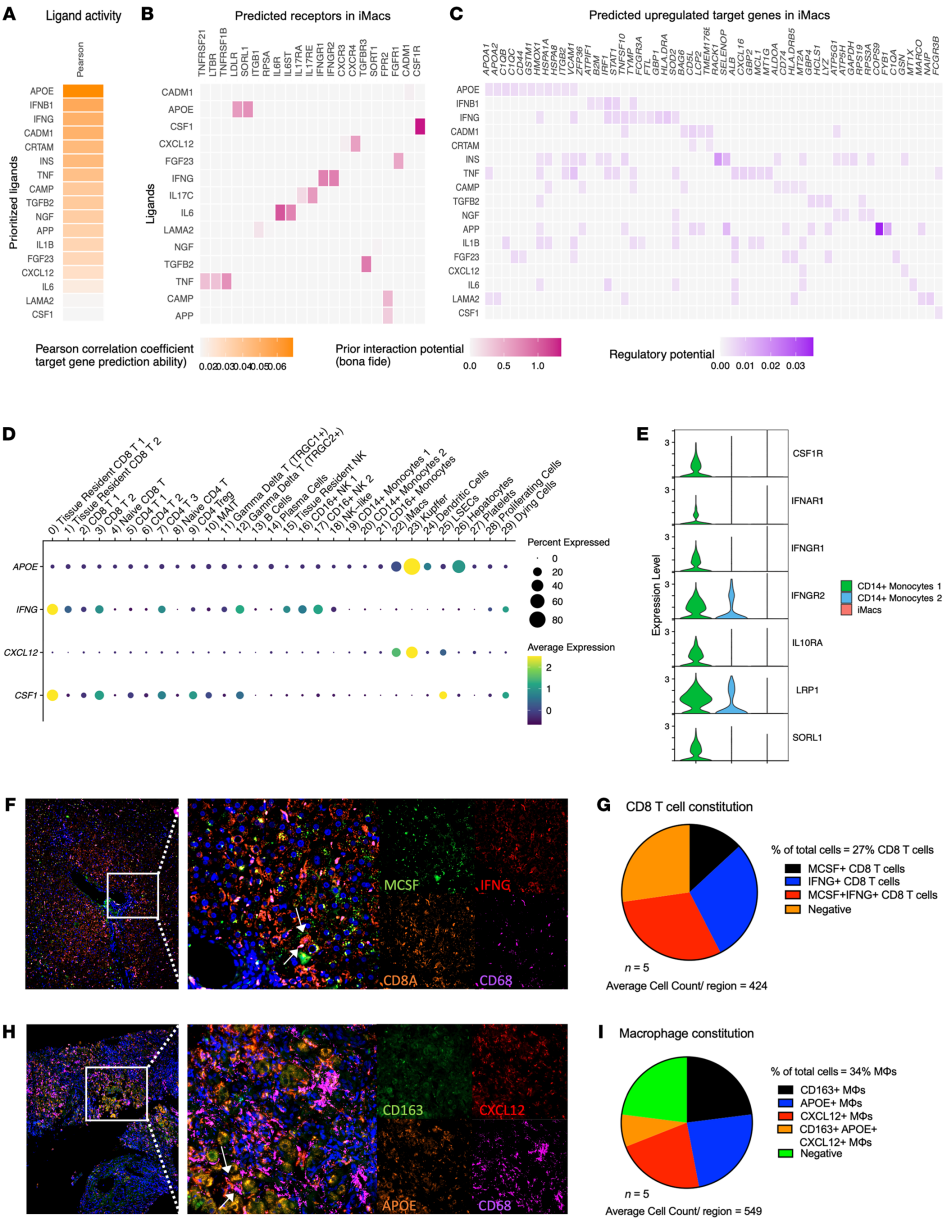
Predicted ligand-receptor interactions during liver inflammation suggest an inflammatory loop between iMacs and CD8^+^ T cells that will serve to guide in vitro monocyte-to-macrophage differentiation. (**A**–**C**) NicheNet analysis of ligand-receptor pairs that induce the differentially expressed gene profile of iMacs during liver inflammation (baseline). (**A**) Potential ligands, (**B**) receptors, and (**C**) target genes that may drive macrophage differentiation and activation at baseline. Only the ligand-receptor interactions that have been previously reported in the literature are included in **C**. (**D**) Dot plot of the NicheNet-predicted ligands for all Seurat clusters. (**E**) Violin plot of the NicheNet-predicted receptors on the iMacs and on both CD14^+^ monocyte clusters. All selected genes have an adjusted *P* value of less than 0.05. (**F** and **H**) RNAscope images depicting colocalization of ligands from the NicheNet analysis in (**F**) CD8^+^ T cells and (**H**) KCs within inflamed liver of patients with CHB (*n* = 5). Original magnification, ×10 (left) and ×40 (right). (**G** and **I**) Percentage of total (**G**) CD8^+^ T cells and (**I**) KCs that expressed NicheNet-suggested ligands at the area of inflammation in patients with CHB (*n* = 5). MΦs, macrophages.

To identify signals driving monocyte differentiation, the NicheNet algorithm (37) was used to infer potential ligand-receptor interactions in iMacs during inflammation. Among the top predicted ligands for iMac differentiation were apolipoprotein E (ApoE), IFN-β, IFN-γ, CXCL12, and CSF1, also known as macrophage CSF (MCSF) (Figure 5, A–C). ApoE was predicted to upregulate *C1QA*, *C1QB*, and *HMOX1* expression. Type I IFN was anticipated to upregulate the IFN-stimulated genes (ISGs) *IRF1* and *STAT1*, whereas type II IFN was predicted to upregulate *GBP1*, *HLA-DRA*, and *SOD2* expression (Figure 5C).

We then identified potential sources of these ligands within our dataset to predict intercellular crosstalk from the scRNA-Seq data. Two key ligands, IFN-γ and CSF1, were mapped to tissue-resident CD8^+^ T cells (cluster 0), whereas ApoE and CXCL12 were highly expressed by KCs (cluster 23) (Figure 5D). Type I IFN transcripts were not detectable within the dataset. Ligand expression in CD8^+^ T cells and KCs was further validated on inflamed tissue sections from patients with CHB using RNAscope (*n* = 5). RNAscope data were quantified using ilastik, a machine learning–based software for cell segmentation and classification (38). Cell counts and colocalization of markers per region were determined using Fiji software. Our analysis confirmed that approximately 30% of CD8^+^ T cells expressed both IFN-γ and MCSF (*CSF1*) in the areas of inflammation (Figure 5, F and G). The expression of CD163, ApoE, and CXCL12 on CD68^+^ macrophages was more complex, probably because TIMD4^+^ macrophages, TIMD4^–^MARCO^+^ macrophages, and TIMD4^–^MARCO^–^ macrophages were grouped in the same “KC” cluster in our scRNA-Seq data. Therefore, the source of these ligands could be from distinct KC subsets around areas of inflammation. We found that CD163, ApoE, and CXCL12 were expressed individually on CD68^+^ macrophages at similar levels (23%, 24%, and 22%, respectively), while the colocalization of all 3 markers was low (8%) (Figure 5, H and I). Still, the colocalization and expression of these ligands by CD8^+^ T cells and KCs with iMacs at areas of inflammation was consistent with the IMC and multiplex IF data described above, providing a local microenvironment for their recruitment, differentiation, and activation (Figure 2, D, H, and I). We also observed a progressive decrease in the receptors for these ligands as monocytes progressed through differentiation stages from monocyte 1 to monocyte 2 to iMac (Figure 5E). Therefore, local signals predicted to be responsible for monocyte differentiation to iMacs are contained within the inflamed liver microenvironment.

### Predicted ligands drive iMac differentiation from blood monocytes.

**Figure 6:**
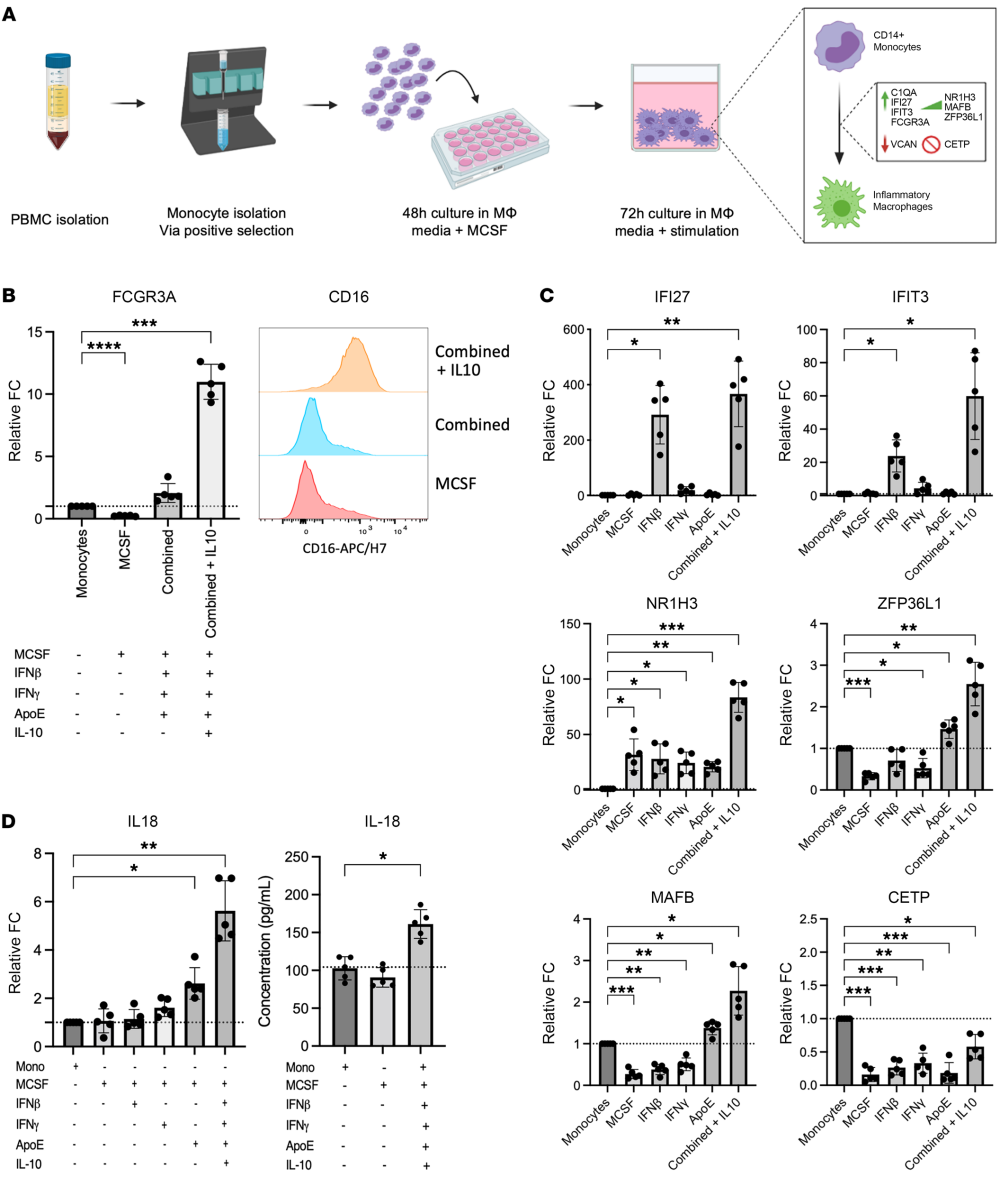
In vitro monocyte-to-macrophage differentiation suggests that type I and II IFN stimulation upregulates key markers from iMacs predicted by scRNA-Seq analysis. (**A**) Workflow used for in vitro monocyte-to-macrophage differentiation using NicheNet ligands with a predicted phenotype. (**B**) Expression of CD16 (FCGR3A) at the transcriptional and protein levels using IL-10 stimulation in addition to NicheNet-predicted ligands. CD16 protein expression was measured from the MFI. (**C**) Real-time quantitative PCR analyses of the relative FC for mRNA expression in differentiated Macs using individual and combined ligands predicted by NicheNet. (**D**) Expression of IL-18 at the transcriptional and protein levels using NicheNet ligands. Combined ligands include MCSF, IFN-β, IFN-γ, and ApoE. Mono, monocytes. *P* values were determined by repeated-measures 1-way ANOVA (*P < 0.05, **P < 0.005, and ***P < 0.001). Results are representative of 5 experiments.

Our analysis predicted that iMacs differentiate from monocytes. Therefore, we evaluated whether we could generate the iMac phenotype from monocytes in vitro (Figure 6A). We used MCSF to prime monocytes for differentiation and then used individual cytokines with the highest predictive potential to induce markers corresponding to the transcriptional phenotypes: ISGs (*IFI27* and *IFIT3*) (33, 34), markers of inflammation (IL-18) (30), *FCGR3A* (CD16), and recent monocyte-to-macrophage differentiation (*NR1H3*, *ZFP36L1*, and *MAFB*) (4, 29, 39). Loss of *VCAN* expression and increased *C1QA* expression were used to validate the transition from monocyte to macrophage (22). *CETP* was used as a negative control to exclude KCs.

Exploratory in vitro experiments revealed that CD16 could not be induced by any of the predicted ligands (Figure 6B and Supplemental Figure 3A). For that purpose, we returned to NicheNet to predict ligands that specifically upregulate CD16. IL-10 had the highest regulatory potential score (Supplemental Figure 3B). In vitro monocyte-to-macrophage differentiation validated the capacity of IL-10 to upregulate CD16 at the transcriptional (Figure 6B) and protein (Figure 6B) levels in the presence of the other predicted cytokines under the condition “combined,” which included MCSF, IFN-β, IFN-γ, and ApoE. The ISGs *IFI27* and *IFIT3* were induced by IFN-β. *NR1H3* was induced by MCSF, whereas ApoE significantly increased the expression of *ZFP36L1* and *MAFB* (Figure 6C). The combination of all the predicted cytokines had a synergistic effect on the upregulation of each of the predicted genes (Figure 6C and Supplemental Figure 3C). *CETP* was not induced under any condition, and MCSF decreased *VCAN* and increased *C1QA* expression (Figure 6C and Supplemental Figure 3A).

Once the differentiation conditions for iMacs were defined, their capacity to produce IL-18 was assessed. IL-18 plays a key role in tissue damage during infections and chronic inflammatory diseases and distinguished iMacs from KCs (Figure 2C) (40). IL-18 was significantly induced at the transcriptional level by ApoE alone and further enhanced by the combined cytokine cocktail (Figure 6D). Consistent with transcriptional activation, we also measured the increase in IL-18 protein in cell culture supernatant (Figure 6D). Further evaluation revealed that the differentiated iMacs represented a unique state between M1 and M2 in vitro–differentiated macrophages. They expressed M1 markers, such as HLA-DR and CD40, but lacked CD86 and expressed the M2 marker CD16, but lacked CD206 and CD209 (Supplemental Figure 4A).

At the functional level, iMacs were largely inflammatory by their release of cytokines such as IL-1β, IFN-α*2,* MCP1, IL-8, IL-18, and IL-23, but also secreted IL-10 (Supplemental Figure 4B). iMacs differed from M1 macrophages by their reduced production of TNF-α, IL-6, and IL-12p70 upon TLR stimulation (Supplemental Figure 4C). The in vitro–induced iMacs accumulated succinate and not α-ketoglutarate (α-KG), similarly to M1 macrophages, suggesting a broken TCA cycle (Supplemental Figure 5A). Furthermore, the gene expression levels of *GLUT1* and *NOS2* aligned with a profile of increased glucose transport for ATP production from glycolysis and impairment of the electron transport chain, which affects oxidative mitochondrial respiration, via accumulation of NO. Additionally, the in vitro–induced iMacs did not express isocitrate dehydrogenase (*IDH2*) to promote the conversion of isocitrate into α-KG for its accumulation, nor did it convert pyruvate into lactate via lactate dehydrogenase (*LDHA*), as observed in M2 macrophages (Supplemental Figure 5B) (41–44). Overall, the in vitro–induced iMacs displayed a profile resembling M1 macrophages (45–47). Therefore, we could validate the differentiation pathway from monocytes to iMacs predicted by the ligand-receptor interaction analysis. The iMacs displayed a phenotypic profile that shared characteristics with both M1 and M2 macrophages but were inflammatory in terms of their cytokine production profile.

### iMac transcriptional signatures remain detectable after long-term suppression of liver inflammation by antiviral therapy.

**Figure 7:**
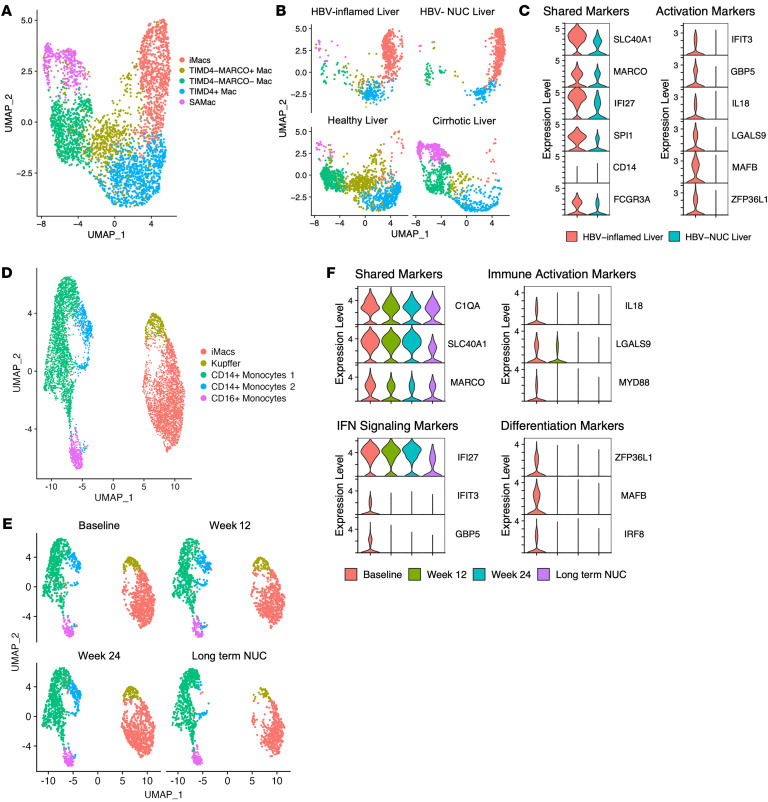
Single-cell-level comparison of iMacs during HBV-related inflammation with healthy livers, cirrhotic livers, and livers of patients with CHB on long-term antiviral therapy. (**A**) Clustering of macrophages (Mac) from healthy (*n* = 5), cirrhotic (*n* = 5), HBV-inflamed (*n* = 5), and long-term NUC therapy (*n* = 5) human livers using UMAP dimensionality reduction. (**B**) UMAP dimensionality reduction of liver macrophages by liver condition. (**C**) Comparison of cluster-defining genes for iMacs in the violin plot during liver inflammation and in the absence of liver inflammation. (**D**) Clustering of CD68^+^ cells during different stages of CHB using UMAP dimensionality reduction. (**E**) UMAP dimensionality reduction of CD68^+^ cells by stage of CHB. (**F**) Violin plots of iMac-defining genes by stage of CHB. All selected genes have an adjusted *P* value of less than 0.05.

Animal models indicate that embryonic KCs are replaced by monocyte-derived macrophages after liver damage. We wanted to understand if the iMacs are short-lived or if they remain in the liver, where they could contribute to future inflammatory events. To address this question, we incorporated an additional scRNA-Seq dataset from patients with CHB. These patients had active hepatitis and then received 4 years of nucleos(t)ide analog (NUC) therapy to reduce HBV replication to undetectable levels and normalize their ALT levels (16). The data from this analysis were integrated with the data from Figure 3 (HBV-inflamed, uninfected healthy [ref. 23], and cirrhotic [ref. 13] human livers), and the same 5 macrophage populations were identified (Figure 7A and Figure 3D). Again, iMacs were enriched in the livers of patients with CHB compared with healthy and cirrhotic livers, but it was visually apparent that iMacs from patients with active disease occupied a distinct space within the cluster compared with iMacs from NUC-treated patients (Figure 7B). iMacs from patients with inflamed livers and NUC-treated patients displayed a similar transcriptional profile: *SLC40A1*, *MARCO*, *IFI27*, *SPI1* (PU.1), *FCGR3A* and an absence of *CD14* expression (Figure 7C, left panel). However, the markers of immune activation (*IL18* and *LGALS9*) (30, 31), IFN signaling (*IFIT3* and *GBP5*) (33, 34), and recent monocyte-to-macrophage differentiation (*MAFB* and *ZFP36L1*) (28, 39, 48) were absent in the patients on NUC therapy (Figure 7C, second column). This supports the concept that iMacs populate the liver during inflammation, but once the inflammation resolves, they may remain in a long-lived quiescent state.

Finally, we compared the distribution of precursor monocyte populations and expression of key genes in relation to the duration of NUC therapy to better understand how these changed as liver damage resolved. Myeloid characterization yielded the same previously defined macrophage and monocyte clusters (Figure 7D). Interestingly, CD14^+^ monocyte 2 (blue), which represented the transitional monocyte population in our Monocle analysis (Figure 2D), was almost undetectable in patients on long-term NUC therapy (Figure 7E). We then compared changes in gene expression over time. While phenotypic markers (*C1QA*, *SLC40A1*, *MARCO*) were stable over time, markers of immune activation, IFN signaling, and recent monocyte-to-macrophage differentiation were decreased by week 12 of antiviral therapy and remained undetectable at 24 weeks and in long-term NUC-treated patients (Figure 7F). Taken together, these data support a model in which liver inflammation gives rise to a unique macrophage that is capable of establishing a long-term survival niche in the human liver, poised to contribute to inflammatory events in the future.

## Discussion

Liver inflammation puts 800 million people at risk for cirrhosis (9), 290 million of whom have CHB (49), with macrophages acting as key regulators of inflammation. While we anticipated macrophage activation from our previous analysis of serum markers in patients with hepatitis (19), our data suggest that the implications of macrophage activation are not transient. Monocytes infiltrating the inflamed human liver integrated complex environmental signals to establish a monocyte-derived macrophage population biased toward inflammation. Imprinting a macrophage population whose abundance positively correlated with CD8^+^ T cells responsible for liver damage suggests they could represent the fulcrum, tilting the scale toward progressive tissue damage during CLD.

The concept of trained immunity has been established for innate immune cells that lack cognate antigen-specific receptors (50). Exposure to a pathogen imprints a functional response based on the inflammatory environment that promotes a predefined response upon reexposure. Despite the fact that monocytes are short lived, both bacille Calmette-Guérin (BCG) vaccination and *Candida albicans* infection can induce a state of trained immunity in adults (51, 52). We previously demonstrated that trained immunity occurs at birth in children born to HBV^+^ mothers, with CD14^+^ monocytes biased toward a Th1-mediated response (53). In vitro, monocytes exposed to inflammatory stimuli adapt their transcriptional profile to environmental signals to influence macrophage differentiation, opening a pathway to reshape the liver macrophage landscape (54). Our longitudinal data show that this occurs over an extended period in patients, with the transitional CD14^+^ monocyte 2 population still present after 6 months of antiviral therapy, but absent after long-term antiviral treatment. Disappearance of the transitional monocyte 2 population and stability of the iMac pool after long-term therapy suggest that iMacs are not constantly being repopulated from the blood and suggest that this recruitment occurs upon niche availability caused by KC death. The iMac population may establish the capacity for self-renewal, similar to what was observed after *Leishmania* infection (55).

The imprinted transcriptional profile of iMacs was clearly distinct from that of KCs, which occupied the same inflamed organ. KCs are biased toward an antiinflammatory profile and remove dying cells to prevent local inflammation (2). However, this can limit their ability to become inflammatory and can potentially be exploited by pathogens. Furthermore, KCs have the capacity to produce low levels of IL-10 at steady state and, in the context of chronic disease, can contribute to the exhaustion and depletion of HBV-specific T cells. In contrast, recently recruited monocyte-derived macrophages are poised to become inflammatory and display enhanced antimicrobial capacity (56). However, their fate upon resolution of inflammation, cell death, or engraftment into the tissue has not been well defined. Our data from patients with CHB after long-term antiviral therapy indicate that iMacs use the niche created in the liver during hepatitis to reshape the macrophage population, which may have a lower threshold for future activation.

When we further compared iMacs with macrophages from healthy and cirrhotic livers, the distinction remained. The relatively fewer macrophages in the KC cluster were dispersed across previously identified macrophage phenotypes in healthy and cirrhotic datasets, while iMacs remained separate and highly enriched in the inflamed liver. The distinction extended to spatial localization, with iMacs clustering around inflamed portal areas, while KCs were dispersed throughout the liver lobules. These data point toward a concept of hyper-local regulation by distinct macrophage subsets across the liver lobules. Whether this spatial distribution endures in patients on long-term antiviral therapy remains to be determined, but knowledge of localization and cell-cell interactions could yield strategies to suppress liver damage across different etiologies of CLD or be exploited for immunotherapy in CHB.

The functional profile of iMacs, their dependence on CD8^+^ T cell–derived cytokines, and colocalization with CD8^+^ T cells suggest that they operate in an inflammatory loop. IFN-γ and MCSF were essential factors to induce the iMac phenotype. The iMac phenotype, being similar to that of M1-like macrophages, was associated with inflammatory cytokine production, including production of IL-18. IL-18, in combination with IL-12, activates CD8^+^ T cells to express IFN-γ and FasL in an antigen-independent manner (57). Consistent with these data, we recently demonstrated that tissue-resident CXCR6^+^ CD8^+^ T cells respond to these cytokines to drive antigen-independent hepatocyte killing through FasL (20). This could create a positive feedback loop, where IL-18 stimulation can lead to the release of IFN-γ which in turn regulates IL-18 release from the iMacs (30). This cycle could be broken by the introduction of antiviral therapy, which rapidly suppresses viral replication.

It is also important to note that, because we were not able to characterize iMac function in vivo, the role of iMacs may not be strictly related to CHB pathogenesis. Intrahepatic myeloid cells can induce the formation of intrahepatic myeloid cell aggregates for T cell population expansion (iMATE) in mice (58). iMATEs were shown to support intrahepatic CD8^+^ T cell expansion and clearance of a chronic infection following therapeutic vaccination in the adenovirus-HBV model. Therefore, it may be possible that iMacs not only regulate nonspecific CD8^+^ T cell activation but also contribute to the expansion of exhausted HBV-specific CD8^+^ T cells during immunotherapy.

In addition to tissue-resident CD8^+^ T cells, KCs appear to play a role in orchestrating iMac differentiation. Our IMC data confirmed that KCs were not completely eliminated and remained in the inflamed liver. During inflammation, KCs remained distributed throughout the lobule. Comparison of macrophages between the healthy and cirrhotic liver also highlighted the heterogeneity of the KC cluster in our dataset, which was composed of at least 3 different phenotypes. This suggests that disparate populations of KCs may be responsible for regulating different aspects of inflammation in patients with CHB. At areas of inflammation, 1 subset of KCs seems to regulate monocyte recruitment through CXCL12, whereas another distinct subset may regulate iMacs via ApoE. ApoE is a physiological regulator of lipid homeostasis with the capacity to modulate the immune response against pathogens, such as malaria, mycobacteria, and viruses (59). Its role in monocyte-to-macrophage differentiation was that of an enhancer, inducing *MAFB* and *ZFP36L1* to drive monocyte-to-macrophage transition and work in concert with IFN-γ to enhance IL-18 expression. When put into context, KC activation through danger signals associated with HBV replication could trigger the recruitment and activation of monocytes. Infiltrating monocytes can integrate inflammatory signals from KCs and CD8^+^ T cells, synergistically increasing their inflammatory transcriptional signature (10, 46–47).

We knew that monocyte-derived macrophages were present in the human liver during inflammatory events. However, previous studies in humans captured a snapshot of the liver environment during inflammatory disease because core biopsies were collected to confirm the diagnosis or staging, not the dynamic changes in activation profiles. Therefore, previous studies lacked dynamic longitudinal data demonstrating the transition from inflammatory monocyte–derived macrophages during liver damage to a (potentially) long-lived macrophage poised for reactivation. With this in mind, the role of iMacs in the different stages of CHB, which are defined by different degrees of viral replication and liver damage, require further investigation. We hypothesize that iMacs arise as patients with CHB transition from an immune-tolerant state of high viral load with normal ALT levels to an immune-active state, in which ALT begins to rise. The iMacs become established in the liver, reshaping the inflammatory potential in the liver that may drive progression to cirrhosis or affect the outcome of immunotherapy by providing a liver-restricted niche for CD8^+^ T cell activity. Understanding how the iMac population is established and how it reshapes the intrahepatic environment could provide key insights to manage liver inflammation across etiologies of CLDs and refine approaches for immunotherapy.

## Methods

### Sex as a biological variable.

Sex was not considered as a biological variable.

Full details on the methods used in this study are provided in the Supplemental Methods.

### Statistics.

All data are presented as the result of 5 or 6 independent experiments and are expressed as the mean ± SEM. GraphPad Prism 9.3.0 (GraphPad Software) and R version 4.0.3 were used to evaluate these data. The statistical analysis performed for each experiment is included in the figure legends. A *P* value of less than 0.05 was defined as statistically significant. Outlier analysis was performed using the robust regression and outlier removal (ROUT) method (*Q* = 1%). The number of replicates is indicated in the respective results sections and figure legends.

### Data availability.

The datasets are available in the NCBI’s Gene Expression Omnibus (GEO) repository (GEO GSE216314). Supporting values for all data points shown in graphs and values behind any reported means are included in the Supporting Data Values file.

### Code availability.

R scripts are available from the corresponding author on reasonable request. Any additional information required to reanalyze the data reported in this work is available from the corresponding author upon request.

### Study approval.

This investigator-initiated clinical study (ClinicalTrials.gov NCT04070079) was approved by the University Health Network Research Ethics Board (CAPCR ID: 18-5748). All patients provided written informed consent.

## Author contributions

AJG, HLAJ, and AG designed the study. HLAJ, SF, and JJF enrolled patients for the study. AJG, JJW, and JJF designed experimental methods. JDSC, SN, DT, VS, DM, AM, and AP performed experiments. JDSV, SN, SCK, DT, VS, DC, KMC, and BW performed data analysis. JDSV and AJG wrote the main manuscript and prepared the figures. All authors reviewed the manuscript.

## Supplementary Material

Supplemental data

Supplemental tables 2-3

Supporting data values

## Version 1 04/15/2025

Electronic publication
