# Supplementary material for: Virus-associated inflammation imprints an inflammatory profile on monocyte-derived macrophages in the human liver
Source: J Clin Invest. 2025 Apr 15;135(8):e175241. doi: 10.1172/JCI175241 (PMC11996867; doi:10.1172/JCI175241)
Supplement: Supplemental data [file jci-135-175241-s195.pdf]

Supplementary Information

Lead contact

Further information and requests for resources and reagents should be directed to, and will be fulfilled by, the Lead Contact, Adam Gehring (Adam.Gehring@uhn.ca)

Experimental models and subjects' details

Study design

This was an investigator-initiated, open-label phase 4 study at the Toronto Centre for Liver Disease, Canada. CHB patients with viral load HBV DNA> 2000 IU/mL and liver damage, measured by elevated levels of serum ALT represented as fold increase over normal values (*ULN >1) started therapy with 25 mg daily of TAF for a duration of 48 weeks and were offered to continue therapy after the end of the study. Blood and FNA samples were collected at baseline, week 12 and week 24. Additional blood samples were collected at week 36 and 48.

Chronic hepatitis B patients

11 patients were included in this study to analyze blood and liver FNAs (NCT: NCT04070079). Inclusion criteria were chronic hepatitis B (HBsAg (+) ≥ 6 months); age >18 years; elevated ALT levels, defined as >19 IU/l for females and >30 IU/l for males (with ULN defined as >25 IU/l for females and >35 IU/l for males); HBV DNA >10000 IU/ml for HBeAg (+) and >1000 IU/ml for HBeAg (-) patients; adequate contraception. An

overview of baseline characteristic is given in supplementary table 1. Exclusion criteria were antiviral or IFN treatment in the previous 6 months; immunosuppressive treatment in the previous 6 months; treatment with an investigational drug in the previous 3 months; history of decompensated liver cirrhosis; liver transplantation; co-infection with HCV, HDV, or HIV; other significant liver disease (such as alcoholic or drug-related liver disease, autoimmune hepatitis, hemochromatosis, Wilson's disease or α1 antitrypsin deficiency); estimated glomerular filtration <50 ml/min/1.73m^2 or significant renal disease; α-fetoprotein >50 ng/ml; pregnancy or breast feeding; other significant medical illness that might interfere with the study (e.g. immunodeficiency syndromes or malignancies); substance abuse. Of the 5 patients analyzed by scRNAseq, 4 were male and one was female. Two patients were HBeAg (+), and three patients were HBeAg (-). Due to low numbers, no analysis of sex influence was included (1).

Human donors of peripheral blood mononuclear cells (PBMCs)

5 healthy human donors were included to obtain PBMCs from whole blood. Mean age at the time of donation was 32.1 years (range 23–53 years). 3 patients were male, and 2 patients were female. Due to low numbers, no analysis of sex influence was included. Informed consent was obtained from all subjects.

Methods details

Analysis of blood markers of HBV infection

Markers of HBV infection in patients' blood were measured at baseline, and at week 12 and week 24 after starting antiviral therapy. HBV DNA levels and ALT times ULN were measured using AmpliPrep TaqMan (Roche) and Advia (Siemens), respectively. HBsAg and HBeAg were measured using the Architect assay (Abbott). Measurement of blood markers of HBV infection was done by the Laboratory Medicine Program of Toronto General Hospital/University Health Network. The clinical characteristics of the patients' profiles is listed in Supplementary table 1.

Liver FNA collection

Liver FNAs were collected by a hepatologist. The site of liver puncture for FNA was determined by bedside ultrasound and patients underwent local anesthesia with 2% lidocaine. 22- or 25-gauge needles were used for aspiration of cells as previously described (https://journals.lww.com/hep/Abstract/9900/Single_cell_RNA_sequencing_of_liver_fine_needle.421.aspx). 20,000 cells per sample were subjected to scRNAseq.

Determination of cytokine concentrations from plasma

Detection of sCD163, IL-18 and Galectin-9 from plasma collected from CHB patients were performed using a custom made Human Magnetic Luminex Assay (R&D Systems) at baseline, and 12 and 24 weeks after starting antiviral therapy. The protocol was performed according to the manufacturer's instructions. Data were acquired using a

MAGPIX instrument (Luminex) and concentrations were calculated using xPONENT software (version 4.2).

Tissue staining for IMC

Tissue staining by IMC and image analyses were conducted as previously described (https://insight.jci.org/articles/view/146883), using paraffin-embedded formalin fixed liver tissue slides from chronically HBV-infected patients (28 immune active and 6 immune tolerant subjects).

Gating strategies for FCS files for CD8 T-cells, iMacs and KCs

CSV files exported from R were converted into flow cytometry standard (FCS) files, and ± cutoff values for each marker and sample were manually chosen in FlowJo based on biaxial plots using the CD45 channel for comparison. These cutoff values were then imported into R and used for gating, employing the flowCore and flowWorkspace packages, and subsequent statistical analyses. CD8 T cells were further gated for $CD3^+CD8^+$ while myeloid cells were gated for $CD68^+$, $CD16^+$, and $CD14^+$ cells (Suppl. Fig. 2C). Notably, expression patterns for CD68, CD16, and CD14 were highly colocalized with similar characteristics in quantitative and phenotype analyses. Therefore, we simplified our language and data presentation by using CD68 expression to define hepatic macrophages—defining iMacs as CD68+CD16+CD14- CD45+ cells and KCs as CD68+CD16+CD14+CD45+ cells, whereas CD8 T cells were defined by the expression of CD3 and CD8. Gated immune subsets were then analyzed for phenotype marker

expression (Suppl. Fig. 2D). Results were normalized as hepatic cellular density in counts per mm^2 Region on interest (ROI) and as percentages for each cell subset of interest. Cells were categorized as portal or lobular based on their location within manually annotated regions based on the detection of portal triad (CK19+ bile ducts, CD31+ endothelial cells, and portal vein) in addition to collagen in the absence of hepatocytes.

Cell proximity analysis

FCS files representing each sample ROI from liver biopsies were generated as previously described. For each CD8 T cell, the Euclidean distances to all iMacs and KCs were calculated. CD8 T cells were considered proximal if at least one macrophage of each type was found within a 15 µm radius. Similarly, for each iMac and KC, the number of CD8 T cells within a 15 µm radius was determined.

Multiplex IF

To detect macrophage protein expression, multiplex IF was performed on frozen formalin-fixed paraffin-embedded (FFPE) CHB inflamed liver tissue sections (n= 4). Briefly, slides were sequentially stained with primary antibodies for 20 min at 20°C: CD68, 1:100; CD163, 1:100; and IFIT3, 1:50. Subsequently, slides were stained with secondary antibodies. After which, slides were microwaved to strip of the primary antibodies and apply the next round of staining. These steps were repeated for a total of 6 rounds and an additional round of DAPI staining. Seven color multiplex stained slides were imaged using the Vectra Multispectral Imaging System version 3 (Perkin Elmer). Scanning was

performed at 20X. A spectral library containing the emitted spectral peaks of the fluorophores in this study was created using the Vectra image analysis software (Perkin Elmer). Using multispectral images from single-stained slides for each marker, the library was used to separate each multispectral cube into individual components (spectral unmixing) allowing for identification of all seven marker channels of interest using inForm 2.4.3 image analysis software. Then Machine-learning-based automated image analysis was performed to segmented subcellular compartments, using DAPI stain and extracted the immunohistochemistry (IHC) signals on a per-cell basis.

RNAscope *in-situ* hybridization

RNAscope and IF was performed on 4 μm sequential cut slides from frozen formalin-fixed, paraffin-embedded inflamed liver tissue collected from CHB patients (**n=** 5) by the Toronto Pathology Research Program (Toronto General Hospital) using standard methods. To detect single mRNA molecules with CD68 protein expression, the RNAscope Multiplex Fluorescent Detection kit v2 (Advanced Cell Diagnostics) was combined with the RNA-Protein Co-detection Ancillary kit (Advanced Cell Diagnostics) following the manufacturer's instructions. Samples were prepared for *in situ* hybridization (ISH) through a series of steps including dehydration, deparaffinization, peroxide blocking and target retrieval blocking. Briefly, tissue slides were dehydrated using standard ethanol series and then serially incubated in H202 (Advanced Cell Diagnostics) for 10 min at room temperature, this was followed by RNAscope 1x Target Retrieval agent (Advanced Cell Diagnostics) for 15 min at 99°C. Upon completion, tissue sections were baked dry at 60°C for 5 min. Slides were then incubated in Protease III solution (Advanced Cell Diagnostics)

at 40°C for 30 min. The mRNA in the tissue was hybridized by incubating in RNAscope buffered Z probes for either C1: MCSF or CD163 (Advanced Cell Diagnostics); C2:IFNG or CXCL12 (Advanced Cell Diagnostics); or C3: CD8 or APOE (Advanced Cell Diagnostics) for 2 h at 40°C. Aftwerwards, slides were incubated in RNAscope amplifier components for 30 min at 40°C and conjugated with a fluorescent dye. The attached RNAscope amplifier structure was opened with a channel-specific horseradish peroxidase (Advanced Cell Diagnostics) to allow fluorescent labeling. C1 was conjugated to 1:1500 TSA-520 (Advanced Cell Diagnostics); C2 to 1:1000 TSA-620 (Advanced Cell Diagnostics); and C3 to 1:1000 TSA-570 (Advanced Cell Diagnostics) for 30 min at 40°C and treated with channel specific horseradish peroxidase blocker (Advanced Cell Diagnostics) to close the amplifier structure. Immediately after, tissue sections were incubated with 1:400 anti-CD68 (Agilent Dako) primary antibody solution and was followed by 1:1000 Opal 620 (Akoya Biosciences) secondary antibody solution. Both, primary and secondary antibody solutions were diluted in Co-Detection Diluent (Advanced Cell Diagnostics). Finally, the slides were DAPI stained and mounted with ProLong Gold antifade mountant (Thermo Fisher) for imaging.

Image acquisition, pre-processing and analysis

Images were acquired using a Zeiss Axio Observer Widefield microscope (10x and 40x objectives). Settings were established during the first acquisition for each panel and not modified afterwards. 2-3 ROIs were imaged at 2048 x 2048 pixels for image analysis. To correct for background fluorescence all images were pre-processed using ZEN 3.9 Lite (ZEISS). Images from individual channels were used to segment and classify cells using

ilastik 1.4.0., a machine-learning based automated image analysis software (2). Fiji 2.14.0 was used to quantify co-localization of markers using DAPI stain and marker overlap on nuclei. Percentages of CD8 T cell and macrophage subsets was calculated as an average from the total count/ region of each respective cell type.

10x Genomics sample processing for single-cell RNA sequencing

For scRNAseq, samples were prepared as outlined by the 10x Genomics Single Cell 5' Reagent Kit user guide with a maximum capture target of 3,000 cells. Briefly, after droplet generation, samples were transferred onto a pre-chilled 96-well plate, heat sealed, and cDNA was generated overnight. The next day, cDNA was recovered using Recovery agent (10x Genomics), and then it was purified using a Silane DynaBead mix (ThermoFisher) following manufacturer instructions. Purified cDNA was amplified for 14 cycles, then it was re-purified using SPRIselect beads (Beckman Coulter). cDNA concentration was measured using a Bioanalyzer (Agilent Technologies). 5' cDNA libraries were prepared as outlined by the 10x Genomics' Single Cell 5' Reagent Kit user guide, with modifications to the PCR cycles based on the calculated cDNA library input. Sequencing libraries were generated with unique sample indices for each sample and quantified.

The molarity of each library was calculated based on library size as measured by the Bioanalyzer (Agilent Technologies) and qPCR amplification data. Samples were pooled and adjusted to 10 nM, then diluted to 2 nM. Each 2 nM pool was denatured using 0.1 NaOH at equal volumes for 5 minutes at room temperature. Library pools were further

diluted to a final loading concentration of 14 pM. 150 µl were loaded into each well of an 8-well strip tube and loaded onto a cBot (Illumina) for cluster generation. Samples were sequenced on the HiSeq 2500 (Illumina) system.

Raw sequencing data were aligned to the human genome reference sequence GRCh38 combined with HBV genome and convered to unique molecular identifier (UMI) counts per gene per cell using the CellRanger (10x Genomics) analysis pipeline.

Cell clustering, differential expression, and pathway analysis

The CellRanger-processed filtered feature matrices were analyzed using the Seurat R package (version 3.2.3) (3). The raw digital gene expression matrix (UMI counts per gene per cell) from each sample was filtered, normalized, and clustered. To preserve high quality cells, filtering was performed as follows: Cells with low transcript counts (<200 UMIs) and high mitochondrial transcript ratio (>30%) were removed. Genes that appeared in less than 3 cells were removed as well. Normalization was performed using the scran R package (version 1.18.5) (4). After normalization, data from all samples was integrated and scaled using Seurat. Clustering was performed using standard Seurat package procedures. Principal component analysis (PCA) was used to reduce the number of dimensions representing each cell. The number of components used was determined based on the elbow of a scree plot. A shared nearest neighbor graph was built from distances computed in principal component space with k.param set to 20 PCA dimensions as inputs. Selection of a biologically relevant number of clusters was based on differential expression between neighboring clusters which were identified as the next-

nearest cluster to each cell after the cell's assigned cluster. Clusters were visualized using uniform manifold approximation and projection (UMAP) coordinates of the principal components as implemented in Seurat. Clusters were identified using the Louvain algorithm with a resolution set to 0.9. Cell-type identities for each cluster were determined manually based on canonical transcriptional markers of well-defined parenchymal/ non-parenchymal liver cells. Differential gene expression analysis was performed using the standard area under the curve (AUC) classifier to assess significance. We retained only those genes with a log-transformed fold change of at least 0.15 and expression in at least 25% of cells in the cluster under comparison (Supplementary Table 2). Further clustering was performed in CD68+ clusters to reveal specific myeloid populations. At each stage of this process, data was scaled and PCA was performed to reveal biologically relevant clusters. We removed clusters expressing more than one unique lineage signature in more than 25% of their cells from the dataset as probable doublets.

For the comparison of liver MΦs between different stages of liver disease and healthy human livers described in Figures 3 and 7, scRNAseq datasets were obtained from publicly available reference datasets. Further information on GEO accession number for the datasets is listed in the key resources table. For downstream analysis, individual samples were filtered and normalized using the same parameters previously described. The data was integrated using Seurat, then further clustering was performed on liver MΦs and differential gene expression was determined (Supplementary table 3). Downstream analysis following data integration was performed as previously described.

Pathways enriched in specific clusters in Fig 2D and 2E were elucidated using GSEA (5). Briefly, gene rank lists were compiled in Seurat, and GSEA was performed using the

fgsea R package (version1.16.0). c5.go.bp.v2022.1.Hs.symbols.gmt from [http://www.gsea-msigdb.org/gsea/msigdb/collections.jsp] was used to identify enriched cellular pathways in GSEA analysis.

All heatmaps, UMAP visualizations, violin plots, dot plots and feature plots were produced using Seurat functions in conjunction with the ggplot2 and pheatmap R packages. Information on the respective vignettes is provided in the "additional resources" section.

Pseudotime trajectory analysis

To generate cellular trajectories to infer developmental relationships between Monocytes and the iMacs at the time of liver inflammation we used the monocle R package (v2.18.0) (6). We ordered cells in a semi-supervised manner based on their Seurat clustering and using the top 2,000 highly variable genes as input we sorted cells in pseudotime. Dimensional reduction was performed using the function "DDRTree" to infer potential developmental paths and pseudotime ordering was performed using the "orderCells" function using CD14+ Monocytes (1) as the start point of the trajectory. Differentially expressed genes along this trajectory were identified using generalized linear models via the 'differentialGeneTest' function in monocle. The remaining parameters were default.

Ligand-receptor interaction analysis

To understand cell-cell interactions at the time of liver inflammation and potential upstream signals driving monocyte differentiation into the iMacs we used the nicheNetr R

package (v.1.0.0) (7). Ligand-target prior model, ligand-receptor network, and weighted integrated networks were imported from NicheNet data sets. iMacs were set as receiver/target cell population and all liver FNA clusters were set as potential senders. Baseline was set as the condition at which receiver cells were affected by other cells and week 24 was set as the steady-state condition. Then, potential ligands were ranked based on the presence of their target genes in the gene set of interest. The top 20 ligands and their cognate target genes were inferred and visualized in a heatmap and validated on dot plots. Genes expressed in at least 10% of the cells in one cluster were considered expressed in this cluster. The remaining parameters were default.

Monocyte isolation from human blood

Whole blood was collected from healthy human volunteers into vacutainer tubes containing the anticoagulant ACD (ThermoFisher). Total blood was diluted in a 1:2 ratio with 2% Knockout Serum Replacement (ThermoFisher). Diluted blood was layered over Lymphoprep solution (StemCell) in SepMate-50 tubes (StemCell). Density centrifugation was used to remove red blood cells and the supernatant containing PBMCs was transferred and were washed 2x with PBS (ThermoFisher) + 2% Knockout Serum Replacement (ThermoFisher) to remove remaining Lymphoprep solution and platelets. PBMCs were counted.

CD14+ Monocytes were purified by positive selection using human CD14+ microbeads (Miltenyl Biotec) following manufacturer's instructions. Purity of CD14+ monocytes was assessed and was found to be ≥ 85%. Pure CD14+ Monocytes were resuspended in MΦ

media which included RPMI (ThermoFisher) supplemented with 10% human serum (Sigma-Aldrich), 1% penicillin/streptomycin (Lifetech) and 1% GlutaMAX (ThermoFisher).

In vitro generation of monocyte-derived MΦs

CD14+ Monocytes were plated at a seeding density of 1 x10^6 cells/mL in MΦ media on 48-well untreated plated (ThermoFisher). A parallel incubation was done on 96-well untreated plates (ThermoFisher) for cytokine release measurement upon TLR agonist stimulation. To trigger monocyte differentiation into iMacs, cells were plated at 37°C in a humidified incubator with 5% CO_2 in the presence M-CSF (Goldbio) at 100 ng/mL for 48 hours. Cells were then polarized for additional 72 hours with cytokines predicted by the NicheNet algorithm. Indicated cytokines were used in the following concentrations: IFN-β (Goldbio) at 100 IU/mL, IFN-γ (Goldbio) at 100 IU/mL, ApoE (Sigma) at 5 µg/mL, and IL-10 at 5 ng/mL (Goldbio). Additional cytokines were used for comparison of inflammatory MΦ against canonical M1- (pro-inflammatory) and M2- (anti-inflammatory) MΦ using: IFN-γ (Goldbio) at 100 IU/mL and LPS (Invivogen) at 1 µg/mL for M1 differentiation and IL-10 (Goldbio) at 25 ng/mL and IL-4 (GoldBio) at 25 ng/mL for M2 differentiation after 48 hours in the presence M-CSF (Goldbio) at 100 ng/mL. On day 5, supernatants were collected from the 48-well plates for measurement of cytokine release. This was followed by detachment of adherent MΦs using 10nM EDTA (Sigma-Alrich) in PBS at room temperature for flow cytometry analysis, or for lysis of adherent MΦ for RNA isolation. Further information on reagents used is listed in the key resources table.

RNA isolation and reverse transcription polymerase chain reaction (RT-PCR, qPCR)

Gene transcript levels were determined by qPCR. MΦ total RNA was extracted using the RNeasy Plus Mini Kit (Qiagen), resuspend in 14uL of ultrapure water and quantified using a NanoDrop 2000 (ThermoFisher), which was then converted to cDNA by reverse transcription using the High-Capacity cDNA Reverse Transcription Kit (ThermoFisher). Both protocols were performed following the manufacturer's instructions. Gene expression was quantified with pre-designed TaqMan Fast Advanced Master Mix (Applied Biosystems) on a QuantStudio 6 thermocycler (Applied Biosystems) to investigate markers of iMacs and monocyte-to-MΦ differentiation. Gene expression was then normalized using the 2-ΔΔCq method relative to the expression of the housekeeping gene, GAPDH. Further information on reagents and primers used is listed in the key resources table.

Flow cytometry analysis of PBMC-derived MΦs

For flow cytometry analysis, fixable viability dye eFluor 450 (eBiosciences), 0.05:100 in PBS, was used for staining of dead cells for 10 minutes at room temperature. Subsequently, cells were washed and stained with extracellular antibodies for 30 minutes at 4°C: HLA-DR_BV605, 2.5:100; CD86_BV650, 1:100; CD40_BV711, 2.5:100; CD206_APC, 5:100; CD209_FITC, 2.5:100; and CD16_APC/H7, 2.5:100. All antibodies were diluted in staining buffer (PBS (ThermoFisher) + 1% BSA (Multicell) + 0.1% sodium azide (Sigma-Aldrich)). Cells were washed and permeabilized using Cytofix/Cytoperm (BD Biosciences) for 15 minutes at 4°C and this was followed by staining with intracellular

antibodies for 30 minutes at 4°C: CD68_PE/Cy7, 1:100, which was diluted in PermWash buffer: PBS (ThermoFisher) + 1%BSA (Multicell) + 0.1% sodium azide (Sigma-Aldrich) + 0.1% saponin (Sigma-Aldrich). Further information on antibodies used is listed in the key resources table. Cells were washed 2x, fixed and stored in PBS + 1% Paraformaldehyde (Sigma-Aldrich). The BD FACSSymphony A3 (BD Biosciences) cytometer was used. Data was analyzed with FlowJo (version 10.8.1).

Cytokine release assay

Cytokine protein levels secreted in the supernatant by differentiated MΦs were quantified using the LEGENDplex^{TM} human inflammation panel 1 (BioLegend) for all markers, which included: IL-1β, IFN-*α*2, tumor necrosis factor (TNF)-*α*, MCP-1, Interleukin (IL)-6, IL-8, IL-10, IL-12p70, IL-18, and IL-33. All the experiments were performed following the manufacturer's instructions. The analytes were diluted in a 1:2 ratio before mixing with the samples. The samples were read using the BD FACSSymphony A3 (BD Biosciences) cytometer, and the data were analyzed using the cloud-based LEGENDplex^{TM} Data Analysis Software (BioLegend). Further information on reagents used is listed in the key resources table.

Succinate and alpha-ketoglutarate release assay

Intracellular succinate (Abcam) and α-KG (Sigma-Aldrich) levels were measured using an assay measurement kit according to manufacturer's instructions. Briefly, 1 x 10^6 cells/each assay were harvested and homogenized. Any insoluble material was removed

by centrifugation. The reagents were diluted in a 1:2 ratio before mixing with the standards and samples. The samples were added in duplicates and were read using the Cytation 5 Cell Imaging Multimode colorimetric microplate reader (BioTek) at an absorbance of 450 nm and 570 nm for the succinate and α-KG assay, respectively. Absorbance values and standard values were used to plot a curve and a trendline equation was calculated. Sample absorbance values were then interpolated within the standard curve to determine the amount of succinate and α-KG. Further information on reagents used is listed in the key resource table.

Additional resources

Seurat vignette : https://satijalab.org/seurat/articles/get_started.html

scran vignette: https://rdrr.igo/bioc/scran/f/vignettes/scran.Rmd

fgsea vignette: https://bioconductor.org/packages/release/bioc/html/fgsea.html

Monocle vignette : http://cole-trapnell-lab.github.io/monocle-release/docs/

NicheNet vignette : : https://rdrr.io/github/saeyslab/nichenetr/f/vignettes/seurat_wrapper.md

References

1. Nkongolo S, Mahamed D, Kuipery A, et al. Longitudinal liver sampling in patients with chronic hepatitis B starting antiviral therapy reveals hepatotoxic CD8+ T cells. *Journal of Clinical Investigation*. 2023;133(1). doi:10.1172/JCI158903

2. Berg S, Kutra D, Kroeger T, et al. ilastik: interactive machine learning for (bio)image analysis. *Nat Methods*. 2019;16(12):1226-1232. doi:10.1038/s41592-019-0582-9
3. Stuart T, Butler A, Hoffman P, et al. Comprehensive Integration of Single-Cell Data. *Cell*. 2019;177(7):1888-1902.e21. doi:10.1016/j.cell.2019.05.031
4. Lun ATL, Bach K, Marioni JC. Pooling across cells to normalize single-cell RNA sequencing data with many zero counts. *Genome Biol*. 2016;17(1). doi:10.1186/s13059-016-0947-7
5. Subramanian A, Tamayo P, Mootha VK, et al. Gene set enrichment analysis: A knowledge-based approach for interpreting genome-wide expression profiles. *Proc Natl Acad Sci U S A*. 2005;102(43):15545-15550. doi:10.1073/pnas.0506580102
6. Qiu X, Mao Q, Tang Y, et al. Reversed graph embedding resolves complex single-cell trajectories. *Nat Methods*. 2017;14(10):979-982. doi:10.1038/nmeth.4402
7. Browaeys R, Saelens W, Saeys Y. NicheNet: modeling intercellular communication by linking ligands to target genes. *Nat Methods*. 2020;17(2):159-162. doi:10.1038/s41592-019-0667-5

Suppl. Figure 1. Cell populations extracted from FNAs and sequenced using scRNAseq were consistent and equally distributed across patients. A) UMAP dimensionality reduction corresponding to the one in Figure 1D for each individual patient across 3 timepoints (baseline, week12 and week24). UMAP dimensionality reduction identified 30 clusters. **B)** Each cluster was annotated and assigned to specific cell types using differential gene expression. All selected genes have an adjusted p value < 0.05. FNAs, fine needle aspirates; scRNAseq, single cell RNA sequencing.

Suppl. Figure 2. Characterization and annotation of liver macrophages and CD8 T cells. A, B) Pathway analysis (GSEA) of differentially expressed genes in inflammatory macrophages **(A)** and Kupffer cells **(B)** at baseline versus week 24 of TAF treatment. Pathway enrichment is expressed as the Normalized Enrichment Score (NES) for multiple comparisons. **C)** IMC individual marker assessment for characterization of liver macrophages and CD8 T cells from the inflamed liver of a CHB patient. Portal region outlined in yellow dotted lines. **D)** Voltage determination of positive and negative populations for annotation of liver macrophages and CD8 T cells using individual markers. **E)** Multiplex IF individual marker assessment for characterization of liver macrophages from the inflamed liver of CHB patients (n=4) **(upper row)**; cyan dots represent CD68+CD163+ cells and green dots represent CD68+IFIT3+ cells **(lower row)**.

Suppl. Figure 3. IL-10 stimulation is needed for CD16 upregulation while not impairing the inflammatory potential of the *in vitro* differentiated macrophages. A) Real-time qPCR analyses of relative fold change for mRNA expression on differentiated Macs using individual and combined ligands predicted by NicheNet. **B)** NicheNet analyses predicted IL-10 has the highest regulatory potential score for upregulation of

CD16 (*FCGR3A*) at baseline, compared to week 24 in the iMacs. **C)** The interferon stimulated-gene response and IL-18 is not dampened by IL-10 while preserving CD16 (FCGR3A) expression. “Combined” ligands include MCSF, IFN-β, IFN-γ and Apolipoprotein E (ApoE). *P*values determined by repeated-measures one-way ANOVA, (*P <0.05, **P <0.005, ***P <0.001). Results showed are representative of **n=**5 experiments. MCSF, macrophage colony-stimulating factor; IFN, interferon; iMacs, inflammatory macrophages; Macs, macrophages.

Suppl. Figure 4. The *in vitro* differentiated macrophages resemble canonical M1 macrophages and have the potential to drive inflammation. A) Protein expression of canonical M1 (pro-inflammatory) and M2 (anti-inflammatory) Macs in different types of *in vitro* differentiated Macs. **B)** Inflammatory cytokines released by different types of *in vitro* differentiated Macs, **n=** 6. **C)** Low doses of IL-10 can still dampen the release of inflammatory cytokines of *in vitro* differentiated macrophages **(top row)** compared to M1 macrophages **(bottom row)** even after stimulation with different TLR agonists. *P*-values determined by repeated-measures one-way ANOVA (*P <0.05, **P <0.005, ***P <0.001). Results showed are representative of **n=**5 experiments, unless otherwise specified. FC, fold change; MFI, median fluorescence intensity; M0, M-CSF only stimulated macrophages; M-CSF, macrophage colony-stimulating factor; iMacs, inflammatory macrophages; Macs, macrophages; TLR, toll-like receptor. “Induced iMac” ligands include M-CSF, IFN-β, IFN-γ, Apolipoprotein E (ApoE) and IL-10.

Suppl. Figure 5. The *in vitro* differentiated macrophages resemble canonical M1 macrophages at the metabolic level. A) Succinate and alpha-ketoglutarate (α-KG) accumulation on differentiated macrophages *in vitro.* **B)** Real-time qPCR mRNA

expression of metabolism regulatory genes on Differentiated Macrophages *in vitro.* *P*values determined by repeated-measures one-way ANOVA, (**P* <0.05, ***P* <0.005, ****P* <0.001). Results showed are representative of **n=**5 experiments, unless otherwise specified. MFI, median fluorescence intensity; M0, MCSF only stimulated macrophages; MCSF, macrophage colony-stimulating factor; iMacs, inflammatory macrophages; Macs, macrophages; TLR, toll-like receptor. “Induced iMac” ligands include M-CSF, IFN-β, IFN-γ, Apolipoprotein E (ApoE) and IL-10.

Resource Tables

**REAGENT or RESOURCE**	**SOURCE**	**IDENTIFIER**
**Antibodies: Flow cytometry**		
Mouse anti-human monoclonal CD68-PE/Cy7 (Y1/82A)	BD Biosciences	Cat# 565595, RRID: AB_2739298
Mouse anti-human monoclonal HLA-DR_BV605 (L243)	BioLegend	Cat#307640, RRID: AB_2561913
Mouse anti-human monoclonal CD86_BV650 (2331)	BD Biosciences	Cat# 563412, RRID: AB_2744456

Mouse anti-human monoclonal CD40_BV711 (5C3)	BD Biosciences	Cat# 563397, RRID: AB_2738181
Mouse anti-human monoclonal CD206_APC (19.2)	BD Biosciences	Cat# 561763, RRID: AB_398476
Mouse anti-human monoclonal CD209_FITC (DCN46)	BD Biosciences	Cat# 561764, RRID: AB_394122
Mouse anti-human monoclonal CD16_APC/Cy7 (3G8)	BD Biosciences	Cat# 560195, RRID: AB_1645466
**Chemical, peptides, and recombinant proteins**		
Phosphate buffered saline	ThermoFisher Scientific	Cat# 14190-144
RPMI Medium 1640	ThermoFisher Scientific	Cat# 22400-097
Human Serum	Sigma-Aldrich	Cat# H3667-100mL
Lymphoprep	Stemcell	Cat# 07851

KnockOut Serum	ThermoFisher Scientific	Cat# 10828-028
GlutaMAX (100x)	ThermoFisher Scientific	Cat# 35050-061
Penicillin/ Streptomycin	Lifetech	Cat# 15070063
EDTA	Sigma-Aldrich	Cat# 03690-100ML
Bovine serum Albumin	Multicell	Cat# 800-095-CG
Saponin	Sigma-Aldrich	Cat# 84510-100G
Sodium azide	Sigma-Aldrich	Cat# S2002-100G
Paraformaldehyde	Sigma-Aldrich	Cat# 158127-100G
Cytofix/ cytoperm	BD Biosciences	Cat# 51-2090KZ
Fixable Viability Dye eFluor 450	eBiosciences	Cat# 65-0863-14
TaqMan fast advanced master mix	Applied Biosystems	Cat# A44360
Armadillo 384 well PCR plates	Applied Biosystems	Cat# AB2384Y

Red Blood Cell Lysis Buffer 10x	BioLegend	Cat# 420301
MCSF	Goldbio	Cat# 1120-09-100
IFN-Beta1b	Goldbio	Cat# 1160-05-2
IFN-Gamma	Goldbio	Cat# 1160-06-20
ApoE3	Sigma-Aldrich	Cat# SRP4696-500UG
IL-10	Goldbio	Cat# 1110-10-2
IL-4	Goldbio	Cat# 1110-04-5
**Critical commercial assays**		
CD14+ microbeads	Miltenyi	Cat# 130-050-201
RNeasy mini kit	Qiagen	Cat# 74106
High-capacity cDNA Reverse transcription kit	Applied Biosystems	Cat# 43-688-14
Legendplex™ human inflammation panel 1 (13-plex) with V-bottom plate	BioLegend	Cat# 740809

Custom Luminex Bead Assay	R&D Systems	N/A
Alpha-ketoglutarate Assay Kit	Sigma-Aldrich	Cat# MAK054-1KT
Succinate Assay Kit (Colorimetric)	abcam	Cat# AB204718
RNAscope Multiplex Fluoresecent Kit v2	ACD	Cat# 323100
RNA-Protein co-detection Ancilliary Kit	ACD	Cat# 323180
scRNAseq 5' v2	10x genomics	N/A
**Oligonucleotides: TaqMan qPCR probe information**		
C1QA	ThermoFisher Scientific	Hs00381122_m1
VCAN	ThermoFisher Scientific	Hs00171642_m1
FCGR3A	ThermoFisher Scientific	Hs02388314_m1
IFI27	ThermoFisher Scientific	Hs01086370_m1

IFIT3	ThermoFisher Scientific	Hs00155468_m1
IL18	ThermoFisher Scientific	Hs01038788_m1
NR1H3	ThermoFisher Scientific	Hs00172885_m1
ZFP36L1	ThermoFisher Scientific	Hs00245183_m1
MAFB	ThermoFisher Scientific	Hs00534343_s1
CETP	ThermoFisher Scientific	Hs00163942_m1
SLC2A1	ThermoFisher Scientific	Hs00892681_m1
NOS2	ThermoFisher Scientific	Hs01075521_m1
LDHA	ThermoFisher Scientific	Hs03405708_m1
IDH2	ThermoFisher Scientific	Hs00953881_m1

GAPDH	ThermoFisher Scientific	Hs02758991_g1
**Multiplex IF: probe information**		
Mouse anti-human monoclonal CD68 (KP1)	DAKO	Cat# MO814
Mouse anti-human monoclonal CD163 (10D6)	abcam	Cat# CM353AK
Rabbit anti-human Polyclonal IFIT3	invitrogen	Cat# PA522230
Opal 520	Akoya	Cat# FP1487001KT
Opal 540	Akoya	Cat# FP1494001KT
Opal 570	Akoya	Cat# FP1488001KT
Opal 620	Akoya	Cat# FP1495001KT
Opal 650	Akoya	Cat# FP1496001KT
Opal 690	Akoya	Cat# FP1497001KT
10x Spectral DAPI	Akoya	Cat# FP1490
**RNAscope: probe information**		

Hs-CSF1-C1	ACD	Cat# 313001
Hs-IFNG-C2	ACD	Cat# 310501
Hs-CD8A-C3	ACD	Cat# 560391
Hs-CD163-C1	ACD	Cat# 417061
Hs-CXCL12-C2	ACD	Cat# 422991
Hs-APOE-C3	ACD	Cat# 433091
TSA Vivid 520	ACD	Cat# 323271
TSA Vivid 570	ACD	Cat# 323272
TSA Vivid 650	ACD	Cat# 323273
Mouse anti-human monoclonal CD68 (PG-M1)	DAKO	Cat# M0876
ProLong Gold Antifade mountant	ThermoFisher Scientific	Cat# P10144
**Software and algorithms**		
Cell Ranger	10x Genomics	https://support.10xgenomics.com/singlecell-gene-expression/software/pipelines/latest/what-is-cell-ranger
R version 4.0.3	The R project	https://www.r-project.org/

Seurat	(Stuart et al., 2019)	https://satijalab.org/seurat/
Scran	(Lun et al., 2016)	https://bioconductor.org/packages/release/bioc/html/scran.html
pheatmap	N/A	https://rdrr.io/cran/pheatmap/
ggplot2	(Wickham, 2016)	https://ggplot2.tidyverse.org
Fgsea	(Korotkevich et al., 2021)	https://bioconductor.org/packages/release/bioc/html/fgsea.html
Monocle 2	(Qiu, et al., 2017)	http://cole-trapnell-lab.github.io/monocle-release/docs/
Nichenet	(Browaeys et al., 2019)	https://github.com/saeyslab/nichenetr
GraphPad Prism 9	GraphPad	https://www.graphpad.com/
FlowJo	FlowJo, LLC	https://www.flowjo.com
xPONENT	Luminex Corporation	https://www.luminexcorp.com/xponent/#overview
inForm 2.4.3	Akoya Biosciences	https://www.akoyabio.com/phenoimager/inform-tissue-finder/

ZEN 3.9 Lite	ZEISS	https://www.zeiss.com/microscopy/en/products/software/zeiss-zen-lite.html
ilastik 1.4.0	ilastik	https://www.ilastik.org
Fiji	ImageJ	https://imagej.net/downloads
**Additional Databases**		
Healthy Liver scRNAseq data 3’ V2	(MacParland et al., 2018)	GEO accession GSE115469
Cirrhotic Liver scRNAseq data 3’ V2	(Ramachandran et al., 2019)	GEO accession GSE136103
Long term NUC scRNAseq data	(Chua et al., 2024)	GEO accession GSE252863

Supplemental information

Suppl. table 1. Clinical characteristics of patients with chronic hepatitis B that were included in this study.

	**Mean**	**Range**
Age [years]	45.2	29–64
Male/Female [% of all patients]	67/33	
ALT at Baseline [* ULN]	8.9	1.1–21.8

HBV DNA at screening [IU/mL]	3.09×10^7	2.73×10^4–9.97×10^7
HBeAg (+) at Baseline [% of all patients]	44%	

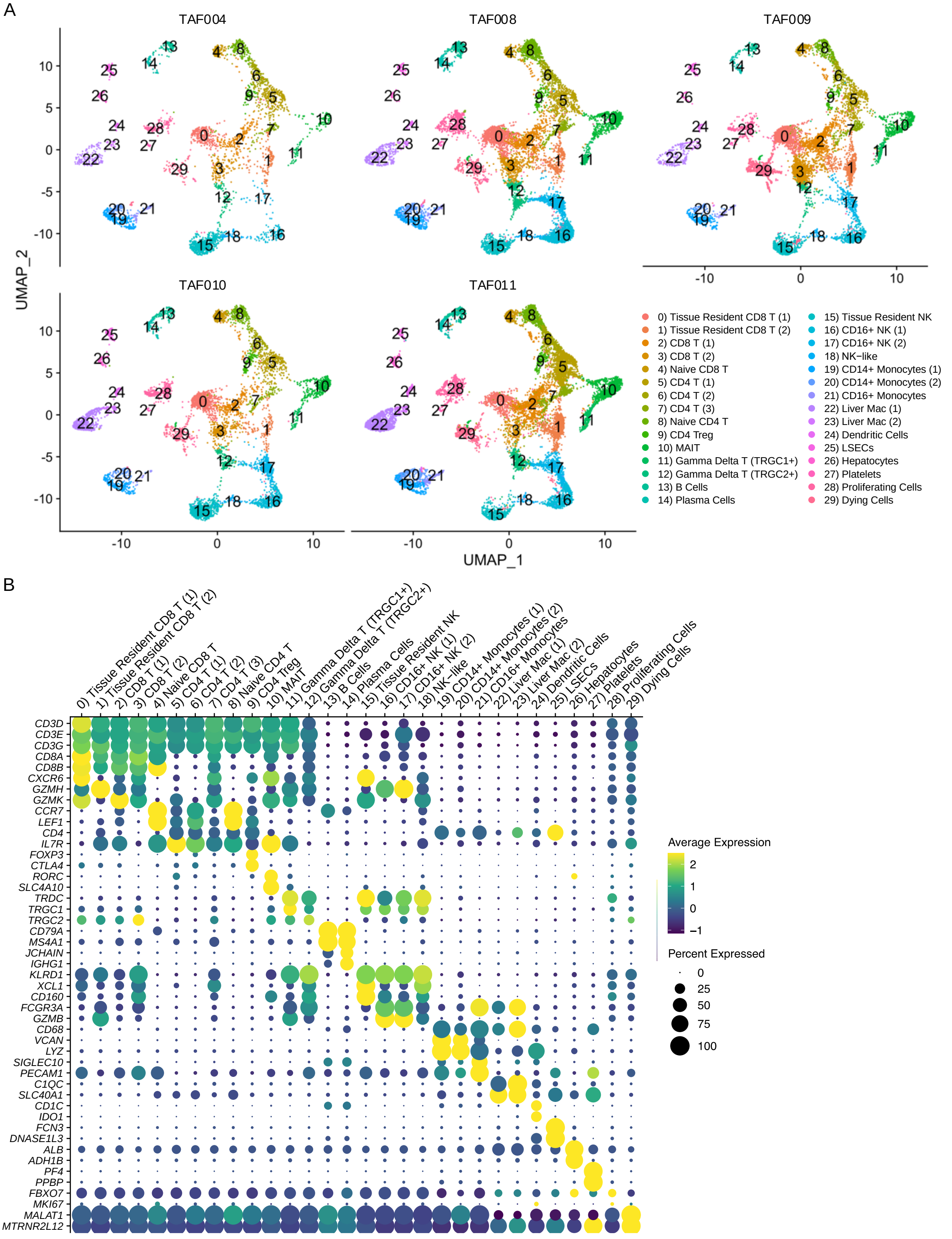

Suppl. Figure 1. Cell populations extracted from FNAs and sequenced using scRNAseq were consistent and equally distributed across patients. A) UMAP dimensionality reduction corresponding to the one in Figure 1D for each individual patient across 3 timepoints (baseline, week12 and week24). UMAP dimensionality reduction identified 30 clusters. **B)** Each cluster was annotated and assigned to specific cell types using differential gene expression. All selected genes have an adjusted p value < 0.05. FNAs, fine needle aspirates; scRNAseq, single cell RNA sequencing.

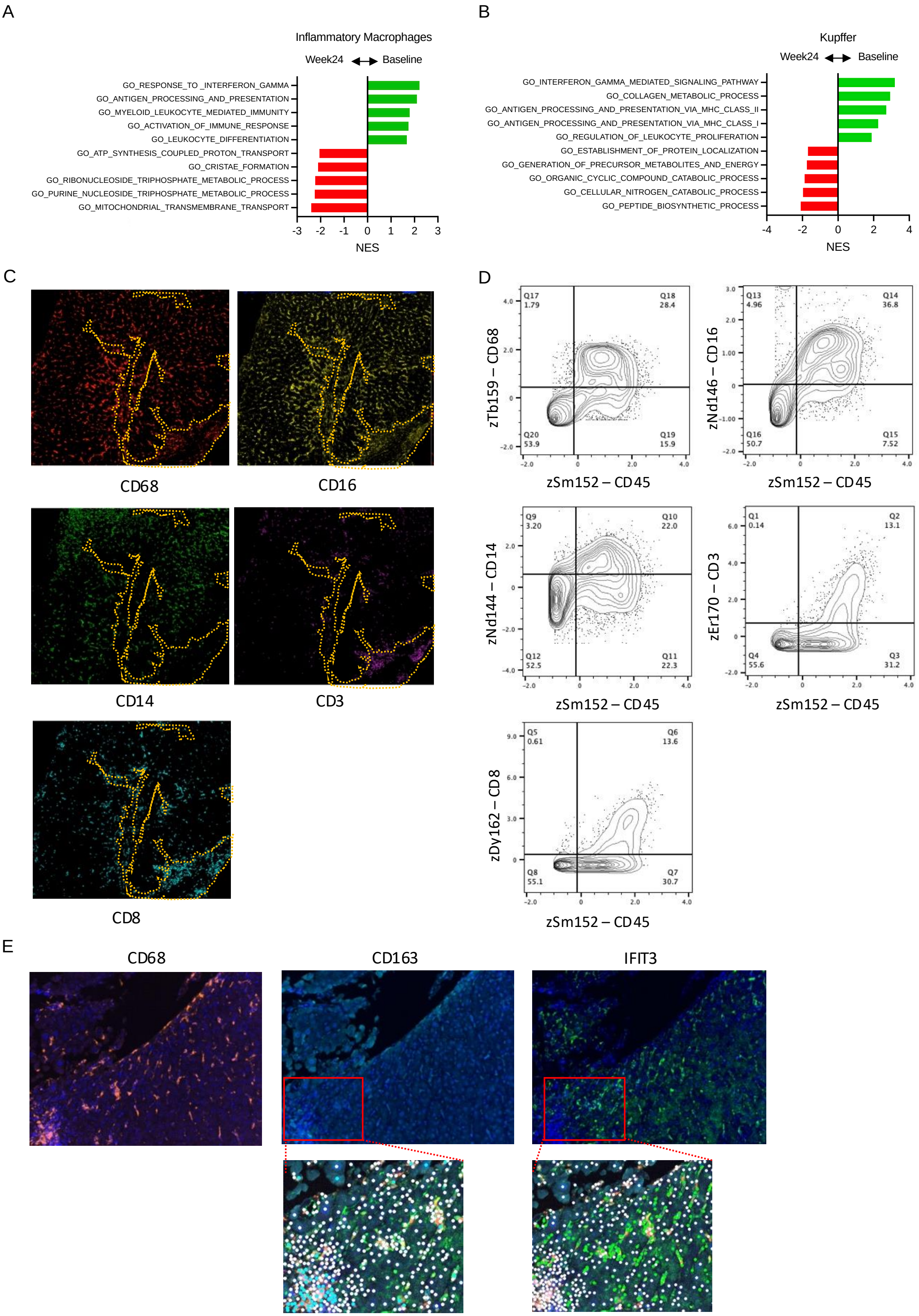
A
Inflammatory Macrophages
Week24
Baseline
GO_RESPONSE_TO _INTERFERON_GAMMA
GO_ANTIGEN_PROCESSING_AND_PRESENTATION
GO_MYELOID_LEUKOCYTE_MEDIATED_IMMUNITY
GO_ACTIVATION_OF_IMMUNE_RESPONSE
GO_LEUKOCYTE_DIFFERENTIATION
GO_ATP_SYNTHESIS_COUPLED_PROTON_TRANSPORT
GO_CRISTAE_FORMATION
GO_RIBONUCLEOSIDE_TRIPHOSPHATE_METABOLIC_PROCESS
GO_PURINE_NUCLEOSIDE_TRIPHOSPHATE_METABOLIC_PROCESS
GO_MITOCHONDRIAL_TRANSMEMBRANE_TRANSPORT
-3 -2 -1 0 1 2 3
NES
B
Kupffer
Week24
Baseline
GO_INTERFERON_GAMMA_MEDIATED_SIGNALING_PATHWAY
GO_COLLAGEN_METABOLIC_PROCESS
GO_ANTIGEN_PROCESSING_AND_PRESENTATION_VIA_MHC_CLASS_II
GO_ANTIGEN_PROCESSING_AND_PRESENTATION_VIA_MHC_CLASS_I
GO_REGULATION_OF_LEUKOCYTE_PROLIFERATION
GO_ESTABLISHMENT_OF_PROTEIN_LOCALIZATION
GO_GENERATION_OF_PRECURSOR_METABOLITES_AND_ENERGY
GO_ORGANIC_CYCLIC_COMPOUND_CATABOLIC_PROCESS
GO_CELLULAR_NITROGEN_CATABOLIC_PROCESS
GO_PEPTIDE_BIOSYNTHETIC_PROCESS
-4 -2 0 2 4
NES
C
CD68
CD16
CD14
CD3
CD8
D
Q17 1.79
Q18 28.4
Q20 53.9
Q19 15.9
zTb159 – CD68
zSm152 – CD45
Q13 4.96
Q14 36.8
Q16 50.7
Q15 7.52
zNd146 – CD16
zSm152 – CD45
Q9 3.20
Q10 22.0
Q12 52.5
Q11 22.3
zNd144 – CD14
zSm152 – CD45
Q1 0.14
Q2 13.1
Q4 55.6
Q3 31.2
zEr170 – CD3
zSm152 – CD45
Q5 0.61
Q6 13.6
Q8 55.1
Q7 30.7
zDy162 – CD8
zSm152 – CD45
E
CD68
CD163
IFIT3

Suppl. Figure 2. Characterization and annotation of liver macrophages and CD8 T cells by IMC. A, B) Pathway analysis (GSEA) of differentially expressed genes in inflammatory macrophages **(A)** and Kupffer cells **(B)** at baseline versus week 24 of TAF treatment. Pathway enrichment is expressed as the Normalized Enrichment Score (NES) for multiple comparisons. **C)** IMC individual marker assessment for characterization of liver macrophages and CD8 T cells from the inflamed liver of a CHB patient. Portal region outlined in yellow dotted lines. **D)** Voltage determination of positive and negative populations for annotation of liver macrophages and CD8 T cells using individual markers. **E)** Multiplex IF individual marker assessment for characterization of liver macrophages from the inflamed liver of CHB patients (**n=** 4) **(upper row)**; cyan dots represent CD68+CD163+ cells and green dots represent CD68+IFIT3+ cells **(lower row)**.

A

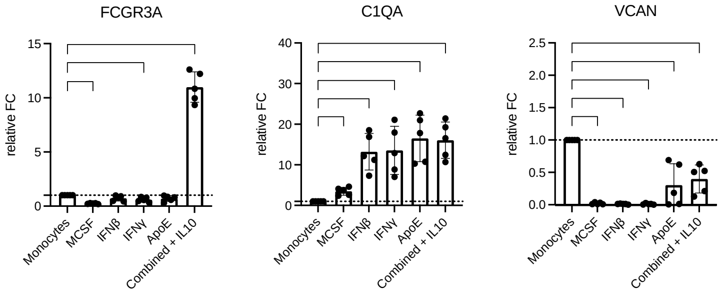

B

Ligand	Regulatory potential score for CD16 in iMacs
IL10	0.004162998

C

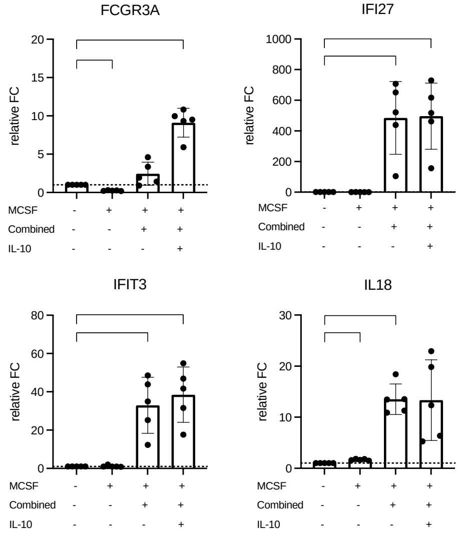

Suppl. Figure 3. IL10 stimulation is needed for CD16 upregulation while not impairing the inflammatory potential of the *in vitro* differentiated macrophages. A) Real-time qPCR analyses of relative fold change for mRNA expression on differentiated macrophages using individual and combined ligands predicted by NicheNet. **B)** NicheNet analyses predicted IL10 has the highest regulatory potential score for upregulation of CD16 (*FCGR3A*) at baseline, compared to week 24 in the iMacs. **C)** The interferon stimulated-gene response and IL18 is not dampened by IL10 while preserving CD16 (FCGR3A) expression. “Combined” ligands include MCSF, IFN-β, IFN-γ and Apolipoprotein E (ApoE). *P*values determined by repeated-measures one-way ANOVA, (**P* <0.05, ***P* <0.005, ****P* <0.001). Results showed are representative of **n**=5 experiments. MCSF, macrophage colony-stimulating factor; IFN, interferon; iMacs, inflammatory macrophages.

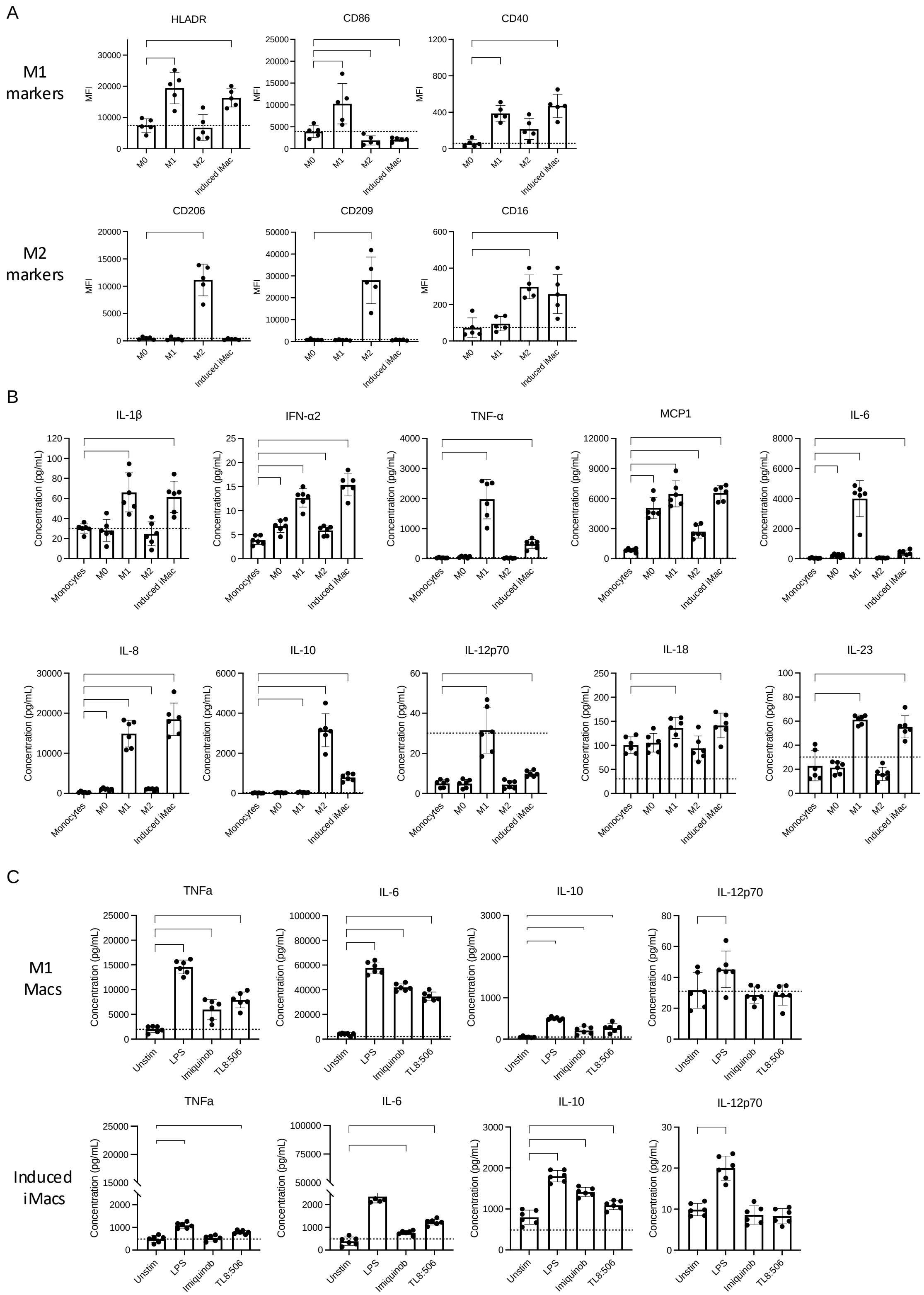
A
M1 markers
HLADR
CD86
CD40
M2 markers
CD206
CD209
CD16
MFI
M0
M1
M2
Induced iMac
B
IL-1β
IFN-α2
TNF-α
MCP1
IL-6
IL-8
IL-10
IL-12p70
IL-18
IL-23
Concentration (pg/mL)
Monocytes
M0
M1
M2
Induced iMac
C
M1 Macs
Induced iMacs
TNFa
IL-6
IL-10
IL-12p70
Concentration (pg/mL)
Unstim
LPS
Imiquinob
TL8:506

Suppl. Figure 4. The *in vitro* differentiated macrophages resemble canonical M1 macrophages and have the potential to drive inflammation. A) Protein expression of canonical M1 (pro-inflammatory) and M2 (anti-inflammatory) macrophages in different types of *in vitro* differentiated macrophages. **B)** Inflammatory cytokines released by different types of *in vitro* differentiated macrophages, **n=** 6. **C)** Low doses of IL-10 can still dampen the release of inflammatory cytokines of *in vitro* differentiated macrophages **(top row)** compared to M1 macrophages **(bottom row)** even after stimulation with different TLR agonists. *P*values determined by repeated-measures one-way ANOVA, (**P* <0.05, ***P* <0.005, ****P* <0.001). Results showed are representative of **n=**5 experiments, unless otherwise specified. MFI, median fluorescence intensity; M0, MCSF only stimulated macrophages; MCSF, macrophage colony-stimulating factor; iMacs, inflammatory macrophages; Macs, macrophages; TLR, toll-like receptor. "Induced iMac" ligands include M-CSF, IFN-β, IFN-γ, Apolipoprotein E (ApoE) and IL-10.

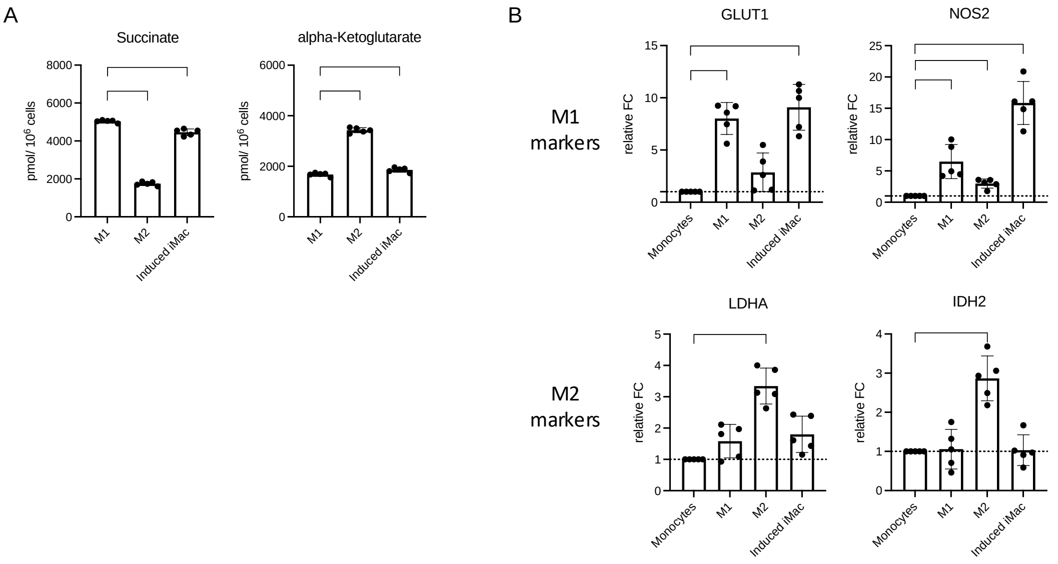

Suppl. Figure 5. The *in vitro* differentiated macrophages resemble canonical M1 macrophages at the metabolic level. A) Succinate and alpha-ketoglutarate (α-KG) accumulation on differentiated macrophages *in vitro.* **B)** Real-time qPCR mRNA expression of metabolism regulatory genes on Differentiated Macrophages *in vitro. P*values determined by repeated-measures one-way ANOVA, (**P* <0.05, ***P* <0.005, ****P* <0.001). Results showed are representative of **n=**5 experiments, unless otherwise specified. MFI, median fluorescence intensity; M0, MCSF only stimulated macrophages; MCSF, macrophage colony-stimulating factor; iMacs, inflammatory macrophages; Macs, macrophages; TLR, toll-like receptor. "Induced iMac" ligands include M-CSF, IFN-β, IFN-γ, Apolipoprotein E (ApoE) and IL-10.
